# Misdirected yet intact TREX1 exonuclease activity causes human cerebral and systemic small vessel disease

**DOI:** 10.1093/brain/awaf085

**Published:** 2025-06-06

**Authors:** Sarah McGlasson, Katy Reid, Anna Klingseisen, Bastien Rioux, Samuel Chauvin, Cathrine A Miner, Joe Holley, Deborah Forbes, Bethany Geary, Jeffrey Kimber, Katrina Wood, Candice Roufosse, Colin Smith, David Kavanagh, Jonathan Miner, David P J Hunt

**Affiliations:** Centre for Clinical Brain Sciences, University of Edinburgh, Edinburgh EH16 4SB, UK; UK Dementia Research Institute at Edinburgh, Edinburgh EH16 4SB, UK; Centre for Clinical Brain Sciences, University of Edinburgh, Edinburgh EH16 4SB, UK; UK Dementia Research Institute at Edinburgh, Edinburgh EH16 4SB, UK; Centre for Clinical Brain Sciences, University of Edinburgh, Edinburgh EH16 4SB, UK; UK Dementia Research Institute at Edinburgh, Edinburgh EH16 4SB, UK; Centre for Clinical Brain Sciences, University of Edinburgh, Edinburgh EH16 4SB, UK; UK Dementia Research Institute at Edinburgh, Edinburgh EH16 4SB, UK; RVCL-S Research Center and Department of Medicine, University of Pennsylvania, Perelman School of Medicine, Pennsylvania, PA 19104, USA; RVCL-S Research Center and Department of Medicine, University of Pennsylvania, Perelman School of Medicine, Pennsylvania, PA 19104, USA; RVCL-S Research Center and Department of Medicine, University of Pennsylvania, Perelman School of Medicine, Pennsylvania, PA 19104, USA; Centre for Clinical Brain Sciences, University of Edinburgh, Edinburgh EH16 4SB, UK; UK Dementia Research Institute at Edinburgh, Edinburgh EH16 4SB, UK; MRC protein phosphorylation Unit, University of Dundee, Dundee DD1 5EH, UK; Crawley Hospital, Sussex and Surrey NHS Trust, Crawley RH11 7DH, UK; National Renal Complement Therapeutics Centre, Newcastle upon Tyne Hospitals, National Health Service Foundation Trust, Newcastle upon Tyne NE1 4LP, UK; Department of Immunology and Inflammation, Faculty of Medicine, Imperial College London, London W12 0NN, UK; Centre for Clinical Brain Sciences, University of Edinburgh, Edinburgh EH16 4SB, UK; National Renal Complement Therapeutics Centre, Newcastle upon Tyne Hospitals, National Health Service Foundation Trust, Newcastle upon Tyne NE1 4LP, UK; RVCL-S Research Center and Department of Medicine, University of Pennsylvania, Perelman School of Medicine, Pennsylvania, PA 19104, USA; Centre for Clinical Brain Sciences, University of Edinburgh, Edinburgh EH16 4SB, UK; UK Dementia Research Institute at Edinburgh, Edinburgh EH16 4SB, UK

**Keywords:** exonuclease, DNA damage, cell cycle, endothelial, vascular dementia

## Abstract

Retinal vasculopathy with cerebral leukoencephalopathy and systemic manifestations (RVCL-S) is an incurable microvascular disease caused by C-terminus truncation of the TREX1 exonuclease. There is a pressing need to understand disease mechanisms and identify therapeutic targets.

We evaluated *TREX1* sequencing data from 469 229 UK Biobank participants together with RVCL-S-related microvascular clinical and imaging outcomes. We show that mono-allelic truncating mutations in *TREX1* require intact nuclease activity in order to cause endothelial disease. Differential proteomics identifies loss of interaction with endoplasmic reticulum insertion proteins such as Guided Entry of Tail-Anchored Proteins Factor 3 as a major consequence of pathogenic TREX1 truncation, and this altered trafficking results in the unregulated presence of enzymatically active TREX1 in the nucleus. In endothelial cells with a patient mutation, mislocalized yet enzymatically active TREX1 causes accumulation of a spectrum of DNA damage. These pathological changes can be rescued by inhibiting exonuclease activity.

In summary, our data implicate exonuclease-dependent DNA damage in endothelial cells as a key therapeutic target in the pathogenesis of RVCL-S.

## Teaser

RVCL is a rare genetic disease of small blood vessels that can cause blindness, dementia, and organ failure. McGlasson *et al.* show that RVCL arises when mutations cause the enzyme TREX1 to mislocalize to the nucleus of endothelial cells, where it causes DNA damage. The misdirected enzyme represents a therapeutic target.

## Introduction

Retinal vasculopathy with cerebral leukoencephalopathy and systemic manifestations (RVCL-S) is a monogenic small vessel disease which causes multiorgan dysfunction, particularly involving the brain and eyes, but also affecting the kidneys, liver and other organs.^1^ RVCL-S is inherited as an autosomal dominant trait, and is caused by mono-allelic mutations in the three-prime repair exonuclease, TREX1.^2^ RVCL-S-causing variants are notable since they all result in frameshift mutations in the C-terminus of the protein, premature protein truncation and mislocalization of the truncated enzyme.

RVCL-S remains untreatable, with progression towards multiorgan failure and brain disease in the early 40s, and death approximately 10 years from diagnosis. While initial decriptions of RVCL-S focused on cerebroretinal involvement, the microvascular bed of almost all major organ systems are involved. These include brain, eyes, limbs, gastrointestinal system, haematopoietic system, bone, thyroid and kidneys.^1,3^ Despite elucidation of the genetic basis of RVCL-S the key aspects of the underlying pathophysiology remain unknown.

TREX1 acts as a negative regulator of the type I interferon response through degradation of intracellular endogenous nucleic acids.^4^ Bi-allelic mutations in *TREX1* cause the type I interferonopathy Aicardi–Goutières syndrome (AGS), leading many to speculate that RVCL-S might also be an interferon-associated disease. Indeed, a number of human studies and mouse models based on RVCL-S mutations have implicated activation of the type I interferon response as a key mediator of disease, either through a loss of cytoplasmic activity or loss of C-terminus binding partners such as oligosaccharyltransferase.^5,6^ These studies have led to clinical trials of agents targeting immune pathways such as aclacinomycin (ClinicalTrials.gov Identifier: NCT02723448), but this approach was halted after the treatment of only four patients. The use of recently developed ultrasensitive digital ELISA has shown that interferon-α (IFN-α) is not elevated in either the blood or CSF of RVCL-S patients, in stark contrast to findings in TREX1-associated AGS (summarized in Supplementary Fig. 1). As such, the evidence to support RVCL-S as an ‘interferonopathic’ disease is weak.^7^

An alternative hypothesis is that the mutant RVCL-S allele causes disease through the production of a toxic TREX1^RVCL^ protein product which is untethered from the endoplasmic reticulum, where the enzyme is usually inserted via the C-terminal transmembrane domain. This active exonuclease would be ubiquitously present in subcellular compartments such as the nucleus, where it would normally be highly spatially and temporally regulated. Importantly, almost all truncations of TREX1 impact subcellular localization, but more extensive C-terminus truncations can also impact exonuclease activity of the protein, for example through disruption of exonuclease domains. Recently published evidence in *Drosophila* and mouse models provide increasing levels of support for this hypothesis, with evidence that DNA damage can accumulate in model systems, with cancer and chromosomal instability newly recognized features of the RVCL disease phenotype.^8^ Specifically, RVCL-associated TREX1 mutants inhibit homology-directed repair (HDR), causing DNA deletions and vulnerability to poly(ADP-ribose) polymerase inhibitors.

This leads to an important directly testable hypothesis in humans. This alternative model predicts that more extensive truncations resulting in both mislocalization and a loss of nuclease function would not cause RVCL-S, or a milder related microvascular phenotype. To date it has not been possible to test this prediction, because it has not been possible to systematically identify individuals with such mutations and examine RVCL-S-related health outcomes in detail. Large population genotype-phenotype studies such as UK Biobank have transformed our ability to look at the pathogenic consequences of single gene mutations at unprecedented scale through linkage of sequencing data with health outcomes. UK Biobank consists of adult volunteers aged 40–69 years at recruitment (2006–2010), which is when major organ disease in RVCL-S become manifest. Thus, our study of *TREX1* in UK Biobank, combined with molecular and cellular analyses informed by these insights, could help resolve some of the key questions around disease pathogenesis.

## Materials and methods

### Analysis of TREX1 truncating variants in the UK Biobank

This research was conducted under UKB Resource application number 93 160. The UK Biobank (UKB) received ethical approval from the North West Multicentre Research Ethics Committee. All participants provided written informed consent. Description of this analysis follows the Strengthening the reporting of genetic association studies (STREGA) statement.^9^ Full methodology for analysis of the UK Biobank data is in the Supplementary material, ‘Methods’ section.

### Patient material

The patient with RVCL provided consent for publication of renal biopsy images, collected as part of routine clinical care and is a participant in the Scottish Regenerative Neurology Tissue Bank/NHS Lothian Bioresource East of Scotland Research Ethics Service Research Ethics Committee 1 15/ES/0094.

### Cell culture

*Trex1* ^−/−^ mouse embryonic fibroblasts (MEFs) were maintained in Dulbecco's Modified Eagle's Medium (DMEM) (Gibco) supplemented with 10% heat inactivated fetal bovine serum (FBS), 1% penicillin-streptomycin-glutamine solution (Gibco) and cultured in 5% CO_2_ and normoxic conditions at 37°C. *Trex1*^−/−^ MEFs were a kind gift from Andrew Jackson/Martin Reijns.

HeLa cells, and all derivative cell lines, were maintained in DMEM supplemented with 10% FBS and 1% penicillin-streptomycin-glutamine solution (Gibco) and cultured in 5% CO_2_ and normoxic conditions at 37°C.

Human brain endothelial cells (hBEC-5i, ATCC-CRL-3245) were maintained in DMEM/F12 (Gibco) supplemented with 10% FBS and 1% penicillin-streptomycin-glutamine solution (Gibco) and 40 μg/ml endothelial cell growth supplement (Millipore) and cultured in 5% CO_2_ and normoxic conditions at 37°C. hBEC-5i endothelial cells were grown on vessels that had been coated with 0.1% gelatin for at least 1 h in a 37°C incubator.

### Stable, inducible expression of EGFP-TREX1 and EGFP-luciferase in HeLa cells

#### Vector construction

The pcDNA5/FRT/TO vector was adapted by Dr M. Harley (A. Jackson laboratory) to include an EGFP cassette and the attR sites required to insert the genes of interest into the vector by Gateway Technology (Invitrogen). Briefly, Dr M. Harley amplified the insert (*EGFP*, chloramphenicol resistance, *ccdB* gene and attR sites) from the pDEST-EGFP vector and linearized the pcDNA5/FRT/TO expression vector with EcoRV (within the multiple cloning site). The In-Fusion system (Clontech) was used to perform the recombination reaction and create the pcDNA5/FRT/TO/GFP.DEST expression vector. Plasmid DNA was amplified by transformation of *Escherichia coli* DB3.1 cells and purified by midiprep (Qiagen).

*TREX1* transgenes were cloned into the pcDNA5/FRT/TO/GFP.DEST expression vector by Gateway Technology and purified by midiprep (Zymo). pENTRY-luciferase was generated by PCR amplification of the *luc2* gene flanked by attB1 sites from the pGL2β vector (obtained from Dr D. J. Klein). Amplification of *luc2* was confirmed by gel electrophoresis and the insert was purified using the QIAquick PCR Purification Kit (Qiagen). *Luc2* was inserted into pDONR221 by BP reaction following the manufacturer’s instructions (Invitrogen).

#### Generation and culture of HeLa cell lines stably expressing EGFP-fusion proteins

HeLa^FTO^ cells containing an *FRT* site and expressing a tetracycline repressor protein were maintained in complete media with blasticidin (5 μg/ml). The pcDNA5/FRT/TO/EGFP-TREX1 vector was cotransfected with pOG44 (expressing flp recombinase) into HeLa^FTO^ using lipofectamine 3000. After 48 h, cells were split into selection media [complete media with blasticidin (5 μg/ml) and hygromycin (400 μg/ml)] to select hygromycin-resistant cells that had incorporated the transgene via flp-mediated recombination. The control cell line expressing inducible EGFP-luciferase was generated previously by Katy Reid.

Gene induction was tested using a tetracycline titration and determined empirically for each cell line (HeLa^FTO^EGFP-TREX1^WT^ 50 ng/ml tetracycline; HeLa^FTO^EGFP-TREX1^V235fs^, HeLa^FTO^EGFP-TREX1^D18N/V235fs^ and HeLa^FTO^EGFP-luciferase 100 ng/ml tetracycline) to induce minimal overexpression of the EGFP-fusion protein.

### EGFP-TREX1 mammalian expression vector construction for transient transfection

Gateway cloning was used to construct mammalian expression vectors. Briefly, the coding sequence of human *TREX1* (ENST00000625293.3) was amplified by PCR to include attB sites and cloned into pDONR221 (Invitrogen) via BP reaction (BP clonase II kit; Invitrogen) to generate an ENTRY vector (pENTRY-TREX1). pEGFP-TREX1 was constructed by recombining the *TREX1* coding sequence from the ENTRY vector into a Gateway converted pEGFP-C2 destination vector (Clontech) via LR reaction (LR clonase II kit; Invitrogen). Minipreps of plasmid DNA (Qiagen) were performed for verification by Sanger sequencing (Source Bioscience). Midipreps of plasmid DNA (ZymoResearch) were performed for mammalian cell transfection.

#### Site-directed mutagenesis

Mutations were introduced into the pEGFP-TREX1 mammalian expression construct by site-directed mutagenesis, as per the manufacturer’s instructions (Q5 Site-Directed Mutagenesis Kit, NEB). Mutations were confirmed by Sanger sequencing.

### TREX1 nuclease activity assay

TREX1 nuclease activity was assayed by transfecting Trex1^−/−^ MEFs with pEGFP-TREX1 wild-type (WT) or variants generated via site-directed mutagenesis) using Lipofectamine 3000 (Invitrogen), as per the manufacturer’s instructions. Whole-cell protein lysate was extracted with radioimmunoprecipitation assay (RIPA) lysis buffer with complete mini protease inhibitor [ethylenediaminetetraacetic acid (EDTA) free, Roche], by incubation for 30 min on ice with occasional agitation. Remaining unlysed cells and debris were removed by centrifugation at 10 000*g* for 10 min at 4°C. Protein lysates were used immediately or stored at −80°C. Protein concentration of the whole-cell lysate was determined by bicinchoninic acid (BCA) assay (Pierce).

Whole-cell lysates (final concentration 100 ng/µl) were incubated with nucleic acid substrate {21-mer single stranded oligo with 3′ fluorescein and an internal DABCYL quencher, [3′fl-intDABCYL-TREX1-21mer, sequence TAGACATTGCCCTCG5AGGTAC (Dabcyl dT at position marked 5, 3′ fluorescein)]; final concentration 200 nM} in reaction buffer [20 mM Tris-HCl, 5 mM MgCl_2_, 2 mM dithiothreitol, 100 μg/ml BSA] in an opaque 96-well plate. Fluorescent measurements were taken with a Clariostar platereader (483-14/530-30; BMG labtech), with temperature control set to 37°C, over a 90 min time course, with a reading every minute.

#### Analysis

**Figure 1:**
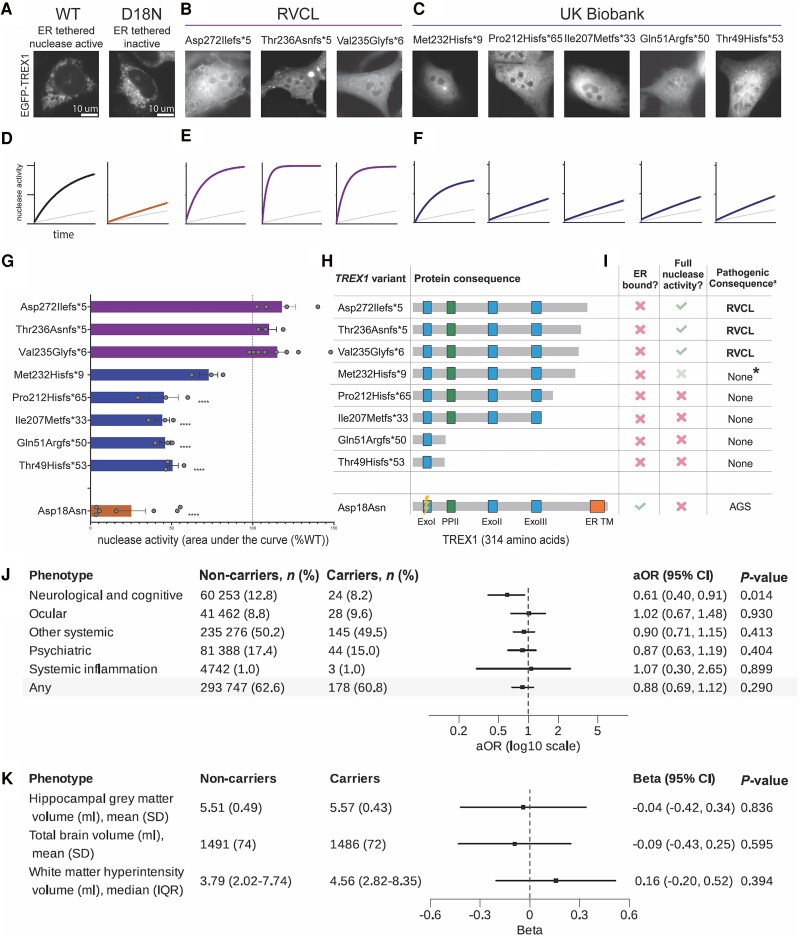
**TREX1 truncating mutations require intact 3′-5′ exonuclease activity to cause RVCL-S.** (**A**) TREX1 function was assayed by expression of an EGFP-TREX1 fusion protein in *Trex1^−/−^* mouse embryonic fibroblasts (MEFs). Wild-type (WT) and exonuclease dead [Asp18Asn (D18N), a dominant negative AGS variant]^10,11^ are shown for comparison. Eight truncating TREX1 variants were introduced into an EGFP-TREX1 expression plasmid in order to express the truncated protein with an N-terminal EGFP fusion. Representative images taken at ×40 from at least three independent experiments are shown. Scale bar = 5 μm. (**B**) Total protein lysate was extracted from transfected MEFs to assay 3′-5′ exonuclease activity. WT activity over time is shown by the black line. Asp18Asn (D18N) activity over time is shown by the orange line. Background lysate exonuclease activity (untransfected cells) is shown by the grey line. (Further details of the nuclease activity assay are in Supplementary Fig. 3) Data presented is from at least three independent experiments, with non-linear curve fit. (**C**) Localization of and (**D**) 3′-5′ exonuclease activity of RVCL-S variants. (**E**) Localization of and (**F**) 3′-5′ exonuclease activity of truncating frameshift variants identified in the UK Biobank. (**G**) Area under the curve was measured with baseline set at time = 0 for UT or D18N, whichever was lowest, and normalized within each experiment to WT = 100%. Data-points show independent experiments, columns show mean. RVCL-S variants in purple, Biobank variants in blue, nuclease dead variant (D18N) in orange. Error bars are standard error of the mean. ****P < 0.0001, One way ANOVA with mixed effects model comparing each data set with WT before data normalization, individual *P*-values are from Dunnett’s multiple comparison test. (**H**) Schematic diagram of protein effects of TREX1 variants. Exo = exonuclease region; PPII = polyproline II; ER TM = endoplasmic reticulum transmembrane. Created in BioRender. McGlasson, S. (2025) https://BioRender.com/k45a920. (**I**) Table summarizing TREX1 variant effects on ER localization, 3′-5′ exonuclease function and clinical disease association. ^a^Pathogenic consequence refers to heterozygotes. ER = endoplasmic reticulum; AGS = Aicardi–Goutieres Syndrome. *M232 variant has slightly reduced nuclease activity, with a possible low penetrance RVCL-S phenotype (see text) Created in BioRender. McGlasson, S. (2025) https://BioRender.com/k45a920. (**J**) Forest plot showing a summary of clinical phenotype associations of truncating TREX1 variants in the UK Biobank. The frequency of 30 RVCL-S-associated outcomes, summarized into five categories, were tested using logistic regression. (Full phenotype outcomes are detailed in Supplementary Fig. 5) (**K**) Forest plot showing neuroradiological associations of truncating TREX1 variants in the UK Biobank. The distribution of three imaging derived phenotypes were tested using linear regression. aOR = adjusted odds ratio; CI = confidence interval; ER = endoplasmic reticulum; IQR = interquartile range; RVCL-S = retinal vasculopathy with cerebral leukoencephalopathy; SD = standard deviation; UT = untreated.

Nuclease assay curves (Fig. 1) from eight independent experiments were assembled as follows. Each of eight independent experiments (tested in triplicate) was individually normalized against WT maximum activity for that experiment to provide a value equating to ‘% of WT nuclease activity’ at each time point. These data were then combined and a curve fitted with non-linear regression. For area under the curve, the baseline was set as the lowest value for either UT or D18N (whichever was lower) and values were assembled from each independent experiment. The statistics were performed on this combined data as detailed in the figure legends. The normalized column graph was assembled by taking WT as the baseline at 100% and comparing each other variant to this.

### Affinity purification of EGFP-fusion protein and putative protein interactions

EGFP-fusion proteins and interacting proteins were purified using GFP-Trap (ChromoTek GFP-Trap Agarose) according to the manufacturer’s instructions. All steps were performed at 4°C. Transgenic HeLa cells (HeLa^FTO^EGFP-TREX1^WT,^ HeLa^FTO^EGFP-TREX1^V235fs^ and HeLa^FTO^EGFP-luciferase) were plated at 5 × 10^6^ cells per 15 cm plate, (four plates total per treatment per cell line) and were treated with tetracycline at the appropriate concentration for 24 h. Plates were then gently washed with PBS and the cells were scraped into 10 ml of cold PBS. Cells were spun at 500*g* for 5 min, the liquid aspirated and the pellet resuspended in 800 μl RIPA buffer with complete mini protease inhibitor. Lysate was incubated for 30 min on ice and spun at 10 000*g* for 10 min at 4°C to remove large debris. Supernatants were transferred to new tubes and stored at −80°C. Three independent experiments were defrosted on ice and the protein was quantified via BCA assay. GFP trap was performed according to the manufacturer’s instructions with the following modifications. Lysate and beads were incubated for 2 h (recommended as the protein has an N-terminal EGFP tag). Samples were eluted in 5% sodium dodecyl sulfate, 50 mM tetraethylammonium bromide, 1× complete mini protease inhibitor, at the request of the UK Dementia Research Institute Proteomics platform. Relative protein quantification by liquid chromatography with tandem mass spectrometry (LC-MS-MS) is detailed in the Supplementary material, ‘Methods’ section.

### Subcellular prediction

Three databases of subcellular proteomics were used, alongside Uniprot, to assign subcellular location to the proteins (for proteins with log_2_fold-change >2 or < −2).^12-14^

### GO term enrichment analysis

GO term enrichment was determined in the STRING protein interaction database (for proteins with log_2_fold-change > 2 or < −2).^15,16^

### Lentiviral transduction of human brain microvascular cells

Lentiviral expression constructs were generated by cloning the EGFP-TREX1^WT^ or EGFP-TREX1^V235fs^ cassette into pLenti6-cppt-CMV-opre. The optimal multiplicity of infection (MOI, viral particles per cell) was determined for hBECs at MOI = 5. Transfection units were calculated using TU_total_ = (MOI × cell number)/viral titre (TU/μl).

hBEC cells were seeded at 4 × 10^5^ cells per well of a 6-well plate and once they were attached, were transduced with virus and polybrene (10 ug/ml). Cells were incubated with virus for 24 h before preparing for imaging or flow cytometry as above.

### Cell cycle analysis by flow cytometry

HeLa^FTO^EGFP-TREX1^WT^, HeLa^FTO^EGFP-TREX1^V235fs^, HeLa^FTO^EGFP-TREX1^D18N/V235fs^ and HeLa^FTO^ were maintained as above. Each cell line was initially seeded into two T25 culture flasks at a density of 22 000 cells/flask in 5 ml of complete culture medium. For each cell line, the EGFP-TREX1 fusion protein expression was induced in one flask by the addition of tetracycline at the optimized concentration. One flask of the HeLa^FTO^ parent cell line was incubated with 100 ng/ml tetracycline as an additional control to determine the effect of tetracycline alone on the cell cycle.

After 7 days of growth the cell lines were 90% confluent. At this stage, the cells were detached from the flask surface with TrpLE (Gibco, Cat. No. 12604), pelleted at 300*g* for 3 min and re-suspended in 10 ml culture medium. The cell lines were again passaged at 22 000 cells per new T25 flask with complete medium (+/− tetracycline). The remaining cells from each flask were pelleted at 300*g* for 3 min. The cell pellets were dissociated and the cells were washed once with 10 ml PBS before pelleting at 300*g* for 3 min. The supernatant was removed from all cell pellets by aspiration to leave ∼100 μl. The cell pellet was resuspended in this volume by flicking the tube. The HeLa cells were fixed by the addition of 900 μl ice-cold 70% EtOH added drop wise while the tube was held on a medium speed vortex. The samples were then stored at −20°C until required. This process was repeated at weekly intervals for 6 weeks.

For flow cytometry of lentivirus transfected hBECS, cells were taken 24 h after transduction and treated as above, except for fixation which was done in 1% paraformaldehyde (PFA).

### Propidium iodide staining for flow cytometry

Cells were thawed on ice for 20 min, with occasional flicking to disperse any cell clumps, and pelleted at 300*g* for 5 min at 4°C. The supernatant was removed by aspiration, the cell pellets were disassociated by flicking the tube, washed once with 10 ml PBS with 1 mM EDTA and pelleted at 300*g* for 5 min at 4°C. The supernatant was removed by aspiration and the cell pellets were disassociated by flicking the tubes. The cells were initially re-suspended in 100 µl of RNase A in PBS/EDTA (final 100 µg) to remove RNA. To stain the DNA, 400 µl of propidium iodide (50 µg) in PBS/EDTA was added to each tube and mixed well, before the sample was transferred into a 5 ml sieved cap tube. Each sample was incubated on ice, protected from light, for at least 5 min before cell cycle analysis.

### Flow cytometry sample acquisition

All samples were acquired on a NovoCyte® Novo Sampler Pro flow cytometer (ACEA Biosciences Inc). First the cells of interest were identified, and the debris removed by plotting the forward scatter height (FSC-H) against the side scatter height (SSC-H). Next the doublet cells were removed from the analysis by plotting the forward scatter area (FSC-A) against the side scatter height (FSC-H). EGFP was detected using 488 nm laser light and a B530/30 bandpass filter. EGFP^-^ and EGFP^+^ cells were separated by plotting SSC-H against B530/30-H. Propidium iodide bound to DNA was detected using 488 nm laser light and a B615/20 bandpass filter. To prevent spectral overlap, the correct level of compensation was determined and applied by plotting B615/20-H against B530/30-H. A cell cycle histogram was plotted and the G1, S and G2 phases of the cell cycle were determined using the cell cycle analysis module within the NovoExpress software (Agilent, https://www.agilent.com/en/product/research-flow-cytometry/flow-cytometry-software) and the Watson Pragmatic algorithm. This reported the percentage of cells in each stage of the cell cycle.

If required, further analysis or confirmation of cell cycle profiles was performed using FlowJo software (v.10.6.1. Becton, Dickinson and Company, NJ, USA) and the Flow Cytometry Standard files exported from the Novocyte.

### Immunofluorescence

#### EGFP-TREX1 localization

*Trex1* ^−/−^ MEFs were seeded onto coverslips at 3 × 10^5^ cells per well of a 6-well dish. MEFs were transfected with EGFP-TREX1 (WT or truncating mutations) 18–24 h after seeding using lipofectamine 3000 according to the manufacturer’s instructions. After 24 h cells were washed with PBS and then fixed in 4% PFA (16% methanol-free PFA, diluted in PBS, Thermo Scientific) for 10 min at room temperature. Cells were washed three times with PBS and permeabilized with 0.2% Triton-X in PBS for 5 min at room temperature. Cell nuclei were stained with 4′,6-diamidino-2-phenylindole (DAPI) [1:6000 in PBS (1 mg/ml)], washed three times with PBS and mounted with Vectashield.

The corrected total cell fluorescence was calculated using a built-in function of FIJI (Imagej.net) to measure integrated density, area of cell and mean fluorescence of image background: CTCF = integrated density—(area of selected cell × mean fluorescence of background readings). The cell area was selected manually for each set of measurements and image background measured in direct vicinity of the cell. Total cell fluorescence was plotted against the number of 53BP1 foci of each cell analysed.

#### DNA damage

Transgenic HeLa cells, lentiviral-transfected hBECs or *Trex1*^−/−^ MEFs were seeded onto coverslips and grown for at least 24 h. Cells were washed with PBS and then fixed in 4% PFA for 10 min at room temperature. Cells were washed three times with PBS and permeabilized with 0.2% Triton-X in PBS for 5 min at room temperature. Cells were washed three times with PBS and blocked in 10% normal goat serum in PBS for at least 1 h at room temperature. Primary and secondary antibody dilutions were performed in antibody incubation buffer [1% bovine serum albumin (BSA) in PBS-T (PBS + 0.1% Tween-20)]. Primary antibody incubations were overnight at 4°C and secondary antibody dilutions were 1 h at room temperature. Primary antibodies used: γH2AX [(Ser139) (20E3) Rabbit mAb Cell Signaling Cat. No. 9718), 53BP1 (rabbit pAb, Novus Biologicals (NB100-904)]. Secondary antibodies: Alexa Fluor-594 (Thermo Fisher Scientific).

Cells were imaged on confocal microscopes Zeiss LSM710 and LSM880 with 20× and 63× Plan-Apochromat oil objectives. To record all DNA damage foci within a nucleus, cells were imaged in 1 µm spaced z-slices using the same settings of laser power and exposure for each set of images. For every data-point of each individual condition at least 50 cells were imaged in at least two biological replicates.

Images were analysed using the ‘find maxima’ command available in FIJI imaging software after maximum projection of the z-stack for each image, to assess the number of γH2AX or 53BP1 positive foci per nucleus. Prominence was set to the highest background value of each individual image. To set foci number in relation to transgene expression of EGFP-TREX1, the number of foci for each nucleus was plotted against EGFP mean intensity divided by background levels over nucleus size as indicated by DAPI staining in maximum projections. Region of interest was drawn manually around each nucleus.

Results from induced/transfected cells were normalized [(x-average control value)/standard deviation] at each time point to that of their non-induced/EGFP only expressing clones grown simultaneously. Each data-point represents the average of the data set for each individual time point and condition.

#### Super-resolution microscopy

Super-resolution microscopy was performed on *Trex1*^−/−^ MEFs transiently expressing EGFP-TREX1^WT^ or EGFP-TREX1^V235fs^. Cells were seeded onto precision coverslips [No. 1.5H (tol. ± 5 μm) (Mareinfeld), cleaned in plasma cleaner] at 3 × 10^5^ cells per well of a 6-well plate, and fixed as previously described in 4% PFA for 15 min. EGFP signal was visualized using an anti-GFP nanobody coupled to ATTO 647N (Chromotek). Calnexin was visualized with Anti-Calnexin antibody (rabbit pAb, ab22595) and a secondary nanobody coupled to ATTO 594. Coverslips were mounted using prolong glass (Invitrogen P36980). Imaging was performed using stimulated emission depletion microscopy (STED) on a Leica SP8.

#### Imaging of chromatin bridges

Chromatin bridges were imaged using DAPI stain (as detailed above) and very high exposure in order to see ultrafine bridges.

### Mice

All protocols for animal experiments were approved by the Institutional Animal Care and Use Committees (IACUC) or Institutional Review Boards at the respective institutions. Full details of transgenic generation is in the Supplementary material, ‘Methods’ section.

### Flow cytometry (mouse cells)

Spleens were kept on ice in RPMI 1640 (Gibco, 11875085) with 2% fetal bovine serum (FBS, Omega Scientific, FB-01). Spleens were digested with 2 ml of RPMI 1640 with 2% FBS, 0.1 mg/ml Dispase I (Sigma, D4818), 2 mg/ml collagenase D (Sigma, 11088858001) and 0.1 mg/ml DNase I (Sigma, DN25) (digestion buffer) was injected into each spleen. Spleens were digested for 30 min at 37°C. Digested spleens were washed in PBS (Gibco, 14190136) supplemented with 2% FBS (FACS buffer). Red blood cells were lysed in ammonium-chloride-potassium lysing buffer (Gibco, A10492-01) for 3 min at room temperature before washing with PBS. For live/dead staining, cells were stained with Zombie NIR (BioLegend, 423106) in PBS for 15 min on ice. Cells were then stained for CD45 (AF700, BioLegend, 30-F11), Ter119 (AF700, BioLegend, TER-119), CD31 (BV421, BioLegend, 390) and podoplanin (PerCP/Cy5.5, BioLegend, 8.1.1) in FACS buffer for 30 min on ice. Fc-mediated interactions were blocked with purified rat anti-mouse CD16/32 (BD Biosciences, 553142) in FACS buffer during surface staining. Cells were then fixed and permeabilized using the FoxP3 Fix/Perm kit (Invitrogen, 00-5523-00) according to the manufacturer’s instructions. Fixed cells were then stained for the HA tag (CST, C29F4) for 30 min on ice before staining with the fluorescently labelled secondary antibody donkey anti-rabbit (AF488, Invitrogen, A-21206) for 30 min on ice. Cells were analysed on an Attune NxT Flow Cytometer (ThermoFisher) and data analysis was conducted in FlowJo v.10 software (FlowJo LLC).

## Results

### RVCL-S mutations cause mislocalization of intact enzyme

We first explored the critical factors required for truncating mutations in *TREX1* to cause microangiopathy in humans. Eight different frameshift mutations which cause truncation from amino acid positions 235 onwards have been reported to cause RVCL-S (Supplementary Fig. 2 and Supplementary Table 1). As anticipated, RVCL-S-causing mutations are exceptionally rare, with only a single individual with a known RVCL-S mutation identified in the UK Biobank (p.Val235GlyfsTer6). We performed recombinant studies of these mutations in *Trex1*^−/−^ MEFs by testing localization of an EGFP-TREX1 fusion protein and TREX1 3′−5′ exonuclease activity, compared with wild-type and a nuclease-inactive variant (D18N) (Fig. 1A and B and Supplementary Fig. 3). We confirmed that the RVCL-S mutations that we tested are associated with widespread mislocalization of the enzyme throughout the cell, but do not affect exonuclease function (Fig. 1C and D). We next examined the clinical consequences of more extensive truncating mutations of sufficient severity to impact exonuclease function.

### *TREX1* truncating variants which erode nuclease activity are not pathogenic

We interrogated the genetic and phenotypic data of 469 229 UK Biobank participants to identify any truncating variant in *TREX1* predicted to cause premature truncation of the protein (Supplementary Table 1). We identified 28 variants in 282 UK Biobank participants (0.06%) which cause extensive truncation, from the start of *TREX1* to amino acid 232 (Supplementary Fig. 4). Our recombinant studies show that mutations in this region cause mislocalization of TREX1, but also significantly impair exonuclease function (Fig. 1E and F).

We compared the health outcomes of truncating variant carriers to those without mutations, with a particular focus on outcomes related to an RVCL-S phenotype and microvascular disease (Fig. 1J and Supplementary Fig. 5). Analysis of linked health records shows that the frequency of six phenotype categories tested using logistic regression was not higher in carriers. Specifically, there was no increase in RVCL-S-associated microvascular disease phenotypes. This included RVCL-S-associated neurological phenotypes (dementia, vascular disease), ocular disease, renal disease, Raynaud’s phenomenon or autoimmunity (Supplementary Fig. 5). We next analysed brain MRI metrics in the 9.3% of individuals where standardized brain imaging was captured. We focused on white matter hyperintensity volume and brain atrophy, since both can reflect accelerated microvascular disease.^17^ We found no difference in white matter hyperintensity volume, total brain volume or hippocampal grey matter volume when tested using linear regression by carrier status (Fig. 1K).

Of note, we identified a frameshift mutation in position 232 that caused a modest decrease in exonuclease activity compared with RVCL-S mutations. Of the five participants in the UK Biobank with this mutation, one was diagnosed with vascular dementia and visual impairment, suggesting this mutation may have pathogenic potential, albeit with reduced penetrance (Fig. 1G–I).

Taken together, our analyses of mono-allelic *TREX1* mutations in the UK Biobank suggest that mutations which cause truncations of sufficient severity to erode the exonuclease activity of TREX1 lose their ability to cause microangiopathic disease. This confirms an important role of intact but mislocalized exonuclease activity in mediating microvascular disease in RVCL-S. We explored this observation further.

### Differential proteomics reveal RVCL-S-truncating mutations cause altered protein interactions

**Figure 2:**
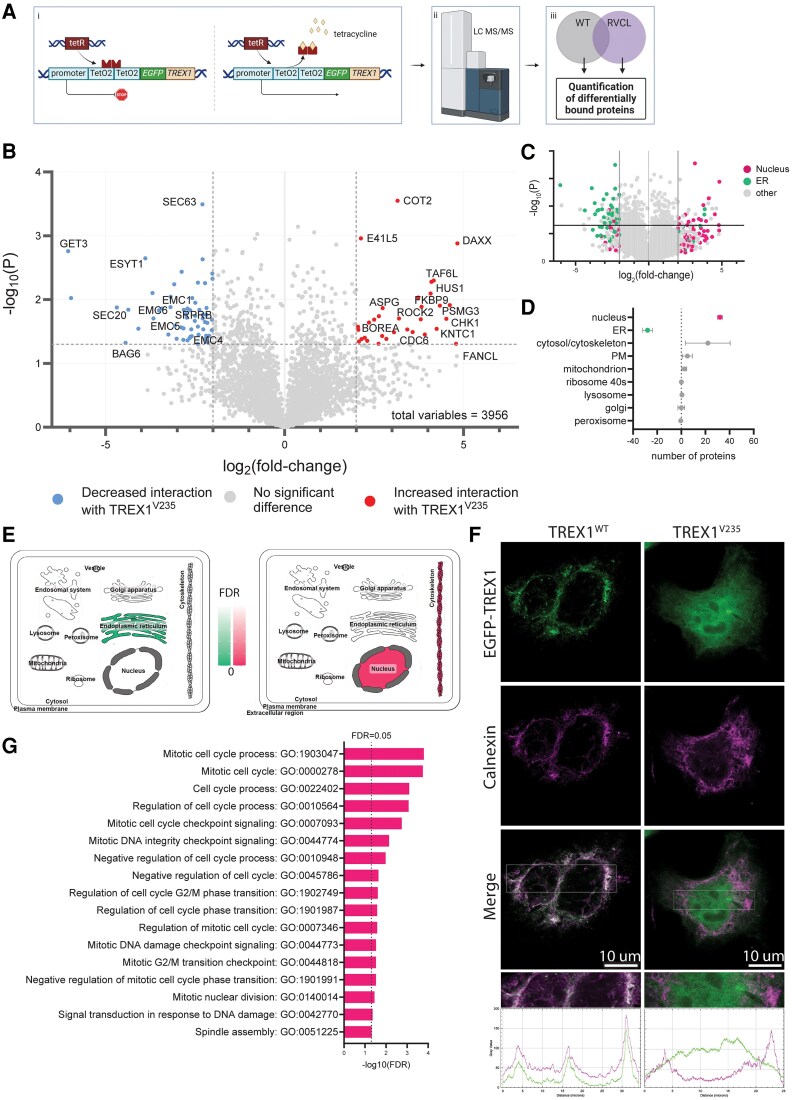
**RVCL-S mutations cause loss of interactions with key components of the post-translational, tail-anchored ER protein insertion pathway**. (**A**) Schematic outline of differential proteomic analysis to identify differential protein interactions between TREX1^WT^ and TREX1^V235fs^. Created in BioRender. McGlasson, S. (2025) https://BioRender.com/b34z593. (**B**) Volcano plot showing the differential protein interactions between TREX1^WT^ and TREX1^V235fs^. The *x*-axis displays the log_2_-fold-change of protein abundance between the conditions, where positive values indicate increased interaction with TREX1^V235fs^ versus TREX1^WT^, and negative values indicate decreased interactions with TREX1^V235fs^ versus TREX1^WT^. The *P*-values are presented on −log10 scale on the *y*-axis. Proteins determined significantly differently bound between TREX1^V235fs^ versus TREX1^WT^ at thresholds *P* < 0.05 are indicated in the uppermost segment. Protein interactions that are more than log_2_(2) increased or decreased are indicated by coloured dots (blue = decreased interactions with TREX1^V235fs^ versus TREX1^WT^; red = increased interactions with TREX1^V235fs^ versus TREX1^WT^). (**C**) Volcano plot of differential protein interactions colour coded by canonical subcellular localization (green = ER; pink = nucleus; grey = other). (**D**) Quantification of the number of proteins that are altered in each subcellular location, in each condition (TREX1^WT^ or TREX1^V235fs^). Data summarized from two independent data sets^12,13^ and Uniprot., (green = ER; pink = nucleus; grey = other). (**E**) Mapping of Go cell component terms associated with decreased (*left*) or increased (*right*) interactions using SubcellulaRVis.^14^ Colour intensity indicates FDR. FDR significance threshold set to 0.01. (**F**) Super resolution (STED) microscopy of EGFP-TREX1^WT^ or EGFP-TREX1^V235fs^, co-stained with calnexin ER membrane protein. Images taken at ×100. Images below quantify the signal intensity of calnexin (magenta) and EGFP-TREX1 (green) across each cell. ImageJ was used to quantify signal intensity across a single cross section. (**G**) GO Process analysis of significant interactions that are increased with TREX1^V235fs^ versus TREX1^WT^. ER = endoplasmic reticulum; FDR = false discovery rate; RVCL-S = retinal vasculopathy with cerebral leukoencephalopathy; STED = Stimulated Emission Depletion; WT = wild-type.

To evaluate further the pathogenic consequences of C-terminus truncation we performed differential proteomics to understand how RVCL-S-causing truncations lead to disease-relevant alterations in protein interactions. We therefore generated human cell lines with inducible, stable expression of *EGFP-TREX1^V235fs^* (V235fs*6, a recognized RVCL-S-causing mutation) or *EGFP-TREX^WT^* and performed tandem mass spectrometry (Fig. 2A). From this experiment, we generated a list of 281 protein interactions that are more than log_2_(2)-fold different between truncated TREX1^V235fs^ and full length TREX1^WT^ (Fig. 2B).

To better understand the spatial subcellular nature of the lost and acquired TREX1^V235fs^ interactions, we parsed data from publicly available data sets and used this in combination with mapping tools to assign the differential interactions to their canonical subcellular locations.^12,13^

Based on canonical subcellular localization, we demonstrated that lost interactions are enriched in endoplasmic reticulum (ER) proteins and gained interactions are enriched in nuclear proteins (Fig. 2C and D). We used SubcellulaRVis^14^ to visualize gene ontology (GO) cellular component enrichment (Fig. 2E).

Differential proteomic analysis identified the protein–protein interaction that was most reduced with TREX1^V235fs^ versus TREX1^WT^ was the interaction with Guided Entry of Tail-Anchored Proteins Factor 3 [GET3 (log_2_-fold-change = −6.04, *P* = 0.0017)]. GET3 is involved in post-translational ER membrane insertion of tail-anchored ER membrane proteins. Analysis of GO process terms further corroborated this reduced interaction, showing a significant enrichment in terms associated with post-translational, tail anchored protein insertion into the ER membrane, and highlighted loss of interaction with other proteins which are involved in the same pathway (such as EMC4, MMGT1, BAG6, EMC6, EMC1) (Supplementary Fig. 6).

We demonstrated the consequence of the failure of ER insertion using stimulated emission depletion (STED) super-resolution microscopy, showing clearly the loss of co-localization between TREX1^V235fs^ and calnexin, an ER membrane protein (Fig. 2F), and demonstrating a clear increase in nuclear localization of TREX1 (Fig. 2F).

GO term analysis from our proteomic data set demonstrates that gained protein interactions with TREX1^V235fs^ are enriched in regulators of DNA damage check point signalling and mitotic cell cycle control (Fig. 2G). Our data suggested interactions between TREX1^V235fs^ and a number of proteins which are known to be involved with DNA damage and cell cycle regulation, including HUS1, CHK1 and DAXX (Fig. 2B). Given these proteomic and STED microscopy results we next explored ways of testing the hypothesis that misdirected TREX1 exonuclease activity in the endothelial cell nucleus causes accumulation of DNA damage.

### Endotheliopathy is a core pathological feature of RVCL-S

**Figure 3:**
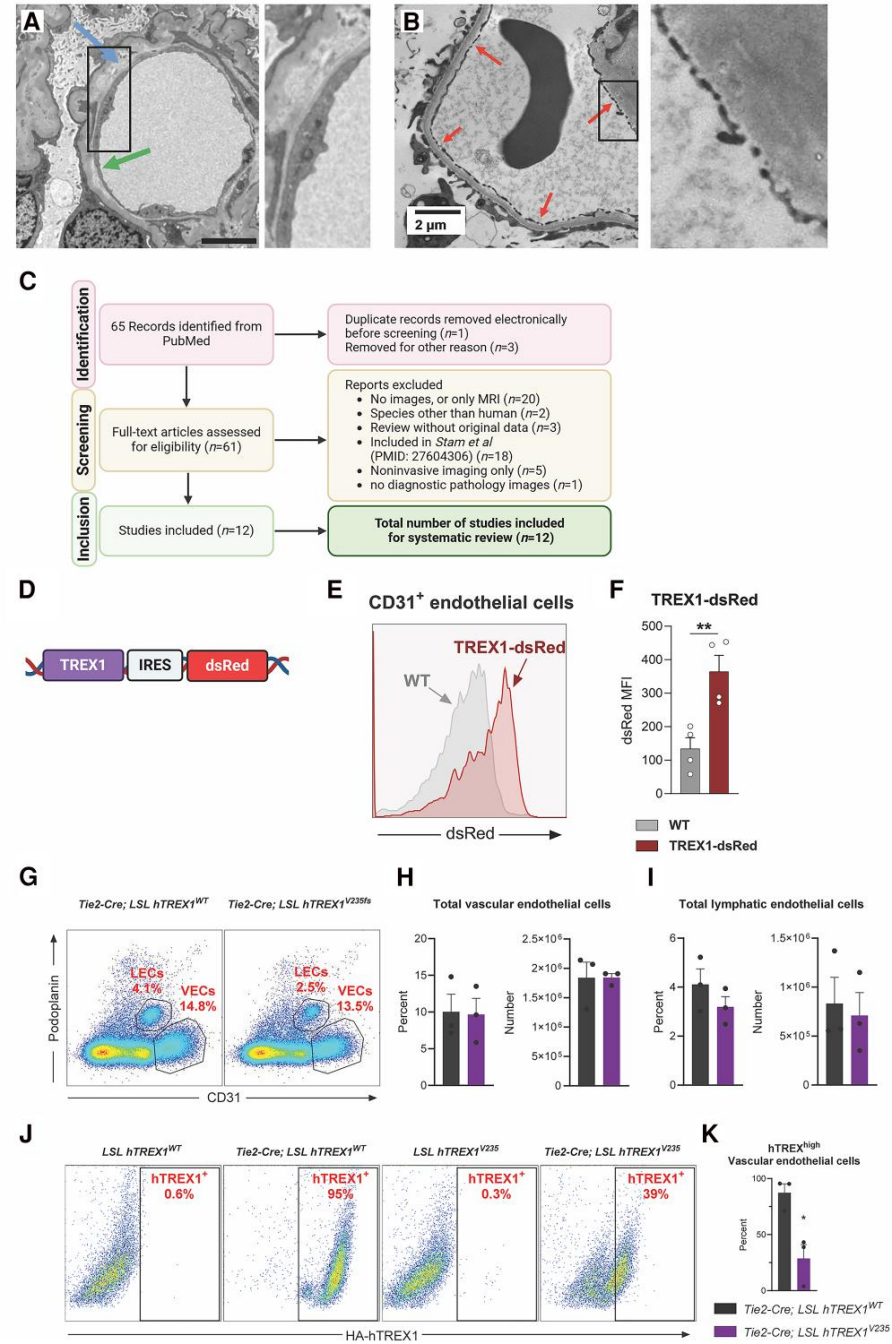
**Endothelial disease is an important component of the microvascular disease observed in RVCL-S.** (**A**) Electron micrograph of microvessel (renal) showing endotheliopathic features such as loss of endothelial cell fenestration (green arrow) and subendothelial lucency (blue arrow). Scale bar = 5 μm. (**B**) For comparison, electron microscopy of normal endothelial cell with fenestrations (red arrows). (**C**) PRISMA pipeline for systematic literature review of RVCL-S pathology.^19^ Created in BioRender. McGlasson, S. (2025) https://BioRender.com/x56u663. (**D**) Schematic of TREX1-dsRed transgenic reporter. These mice express TREX1 and dsRed under the control of the endogenous TREX1 promoter.^8^ Created in BioRender. McGlasson, S. (2025) https://BioRender.com/f91o214. (**E**) Flow cytometry histogram overlaying WT CD31^+^ cells and TREX1-dsRed CD31^+^ cells. (**F**) Quantification of median fluorescent intensity (MFI) of dsRed from independent samples (*n* = 4 per condition). Data-points show individual experiments, columns show mean with standard error of the mean (SEM). *P* = 0.0079, unpaired *t*-test. (**G**) Representative flow cytometry plots of the frequency of vascular endothelial cells (VECs, CD31^+^ popoplanin^−^) and lymphatic endothelial cells (LECs, CD31^+^ popoplanin^+^) in the spleen of Tie2-Cre LSL hTREX1^WT^ or Tie2-Cre LSL hTREX1^V235Gfs^ mice. (**H**) Quantification of the frequency and number of VECs from (**H**). Points indicate data from individual mice (*n* = 3 per group). Columns show mean. Error bars show SEM. (**I**) Quantification of the frequency and number of LECs from (**H**). Points indicate data from individual mice (*n* = 3 per group). Columns show mean. Error bars show SEM. (**J**) Representative flow cytometry plots showing the frequency of hTREX1^+^ vascular endothelial cells via staining the HA tag. (**K**) Quantification of the frequency of hTREX1^+^ VECs from (**J**). Points indicate data from individual mice (*n* = 3 or 4 per group). Columns show mean. Error bars show SEM. *P* = 0.0167 by unpaired *t*-test. RVCL-S = retinal vasculopathy with cerebral leukoencephalopathy with systemic manifestations; PRISMA = Preferred Reporting Items for Systematic reviews and Meta-Analyses; WT = wild-type.

RVCL-S was first described as an endotheliopathy,^18^ TREX1 is expressed in human brain endothelial cells in mouse and human RNA sequencing data sets (Supplementary Fig. 7) and endothelial cells play a prominent role in disease pathogenesis. Consistent with this, we identified pathological evidence of endothelial disease in one of our pateints with a V235fs mutation. Electron microscopy of the renal biopsy showed evidence of endotheliopathic features including loss of endothelial cell fenestration and subendothelial lucency (Fig. 3A and B). To demonstrate further that endotheliopathy is a pathological hallmark of RVCL-S we systematically reviewed all published papers reporting pathological findings in RVCL-S patients, together with an experienced neuropathologist with expertise in microangiopathic disease (Fig. 3C). Endothelial cell disease was identified in 10/12 RVCL-S pathological reports across multiple organs, in particular brain and kidney (Supplementary Table 2). There was insufficient pathological information in the remaining 2/12 studies. We also identified microaneurysym formation, which has been linked to pericyte dysfunction,^20^ in the biopsy from our patient and in a single report from the literature. Overall this review of pathology suggests that endotheliopathy is a core pathological feature of RVCL-S and highlights the need to study the effects of RVCL-S mutations on endothelial cells.

### Endothelial-specific expression of human TREX1^V235fs^ in mice is poorly tolerated

We asked whether we could generate a mouse model of RVCL-S wherein endothelial cells could be induced to express the human RVCL-S allele. First, we tested endothelial expression of TREX1 using the *TREX1-dsRed* mouse model (Fig. 3D). We confirmed that TREX1 is expressed in splenic endothelial cells (Fig. 3E and F). Next, we bred mice which have a floxed-stop, CAG promotor and N-terminal HA-tagged hTREX1^WT^ or hTREX^V235fs^ in the *Rosa26* locus (*LSL-hTREX1^WT^* or *LSL-hTREX1^V235fs^*). We crossed these mice with *Tie2-Cre* animals and quantified vascular and lymphatic endothelial cells to test the effect on total number and percentage (Fig. 3G–I). We found substantially lower expression of HA-hTREX1^V235fs^ than HA-hTREX1^WT^ in the vascular endothelial cells of the spleen, suggesting a survival disadvantage of endothelial cells which activate the mutant allele (Fig. 3J and K). Therefore, to better understand the deleterious effects of expression of the human mutant allele in human brain endothelial cells we studied the consequences of insertion of a mutated patient allele (V235fs) into hBECs.

### Human brain endothelial cells with a mutant RVCL-S allele show increased DNA damage and chromosomal instability

**Figure 4:**
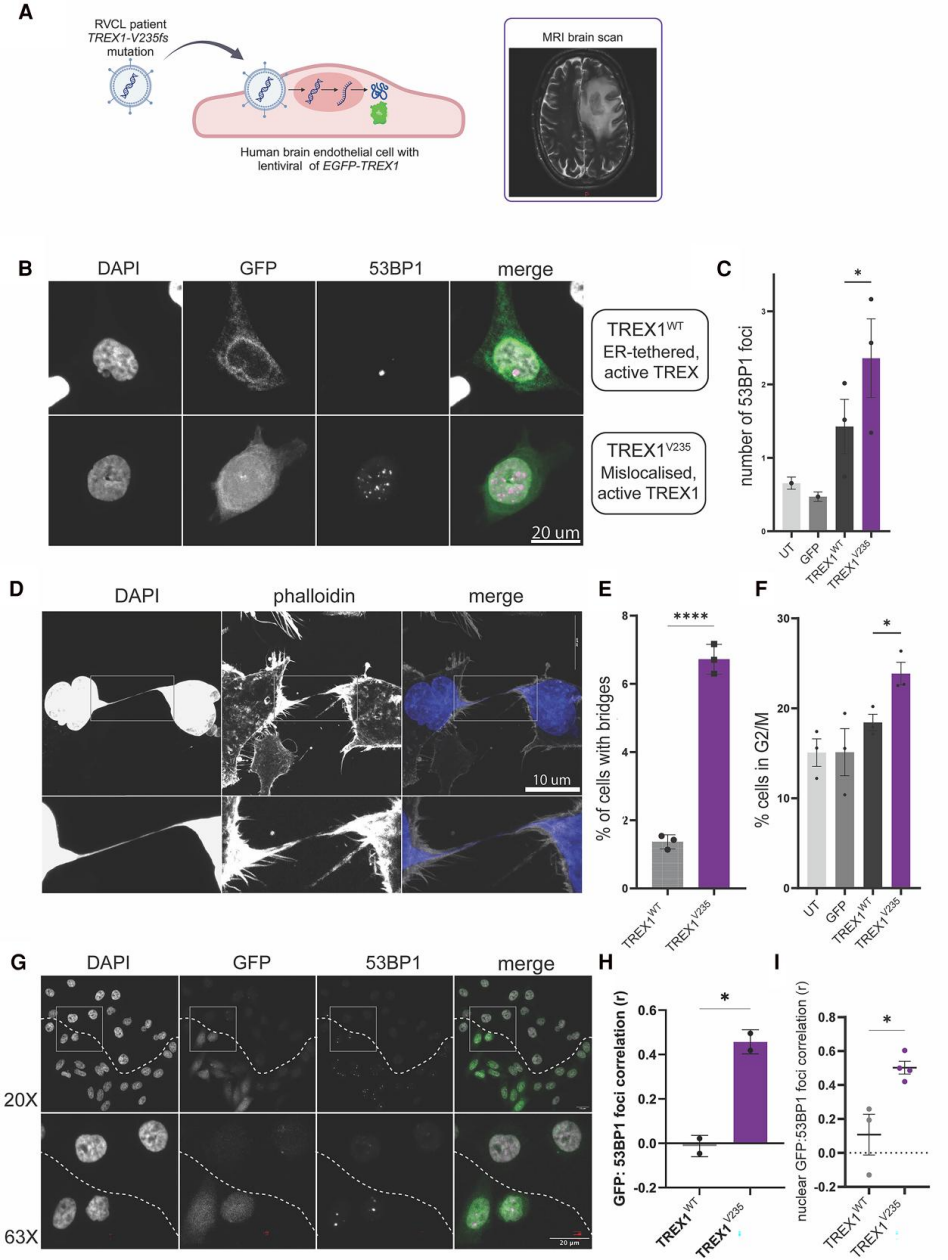
**Human endothelial cells with RVCL-S patient mutations show increased DNA damage, cell cycle defects and increased chromosomal abnormalities**. (**A**) Human brain endothelial cells were transformed using lentivirus to express TREX1 with a patient mutation. The MRI brain scan from a patient with this mutation is shown. Created in BioRender. McGlasson, S. (2025) https://BioRender.com/l89e736. (**B**) Lentiviral expression of EGFP-TREX1 in human brain endothelial cells (hBEC-5i), co-stained with 4′,6-diamidino-2-phenylindole and 53BP1. Illustrative image taken by confocal microscope at ×20 magnification, images shown with digital zoom. (**C**) Quantification of 53BP1 foci in response to lentiviral expression of EGFP-TREX1^WT^, EGFP-TREX1^V235fs^ or an empty EGFP vector. Data-points show three independent experiments and columns show mean. Error bars are standard error of the mean (SEM), *P* = 0.0315, unpaired *t*-test. (**D**) Representative image of chromatin bridge seen in human brain endothelial cells expressing EGFP-TREX1^V235fs^. Confocal images taken at ×63 magnification. (**E**) Quantification of percentage of cells with chromatin bridges. Data-points show three independent experiments and columns show mean. Error bars are SEM, *P* < 0.0001, unpaired *t*-test. (**F**) Quantification of cells in G2/M in response to lentiviral expression of EGFP-TREX1^V235fs^, EGFP-TREX1^WT^ or an empty EGFP vector. Data-points show three independent experiments and columns show mean. Error bars are SEM, *P* = 0.0248, unpaired *t*-test. (**G**) Clonal expression of EGFP-TREX1^V235^ in hBECs. *Top row*: ×20 magnification; *bottom row*: ×63 magnification. Line drawn to indicate GFP^+^ versus GFP^−^ cell clones. (**H**) Quantification of the correlation (Pearson's *r*) between corrected total cell fluorescence (CTCF) of EGFP and 53BP1 foci. *n* = 2 per group, with at least 30 cells counted per experimental group (for full breakdown see Supplementary Fig. 8F and G). Columns show mean. Error bars show standard deviation. *P* = 0.0117, unpaired *t*-test. (**I**) Quantification of the correlation (Pearson's *r*) between nuclear EGFP-TREX1 and 53BP1 foci. *n* = 3 WT, *n* = 4 V235. Points show independent experiments with mean (for full breakdown see Supplementary Fig. 8D and E). Error bars show SEM. *P* = 0.0158, unpaired *t*-test. RVCL-S = retinal vasculopathy with cerebral leukoencephalopathy with systemic manifestations; WT = wild-type.

We studied the consequences of insertion of a mutated patient allele (V235fs) into hBECs. This mutation is the commonest RVCL-S patient mutation and is confirmed to cause microvascular disease in multiple organs (Fig. 4A). We used a lentiviral system to express *EGFP-TREX1^WT^* or *EGFP-TREX1^V235fs^* in hBECs (Fig. 4A).

Based on the broad substrate specificity of TREX1, with affinity for both single stranded DNA and double stranded DNA,^21^ we hypothesized that the increased, apparently unregulated, presence of TREX1 exonuclease in the nucleus would be associated with DNA damage. Indeed, EGFP-TREX1^V235fs^ expressing brain endothelial cells accumulated significantly more DNA damage, measured by 53BP1 foci, compared with EGFP-TREX1^WT^ or EGFP-only expressing cells (Fig. 4B and C). In addition to 53BP1 foci, we also observed an increased frequency of chromosomal abnormalities, including chromatin bridge formation, in endothelial cells expressing EGFP-TREX1^V235^ (Fig. 4D and E). We also tested the effect of EGFP-TREX1^V235fs^ expression on the cell cycle by measuring the proportion of cells in each cell cycle phase using flow cytometry. TREX1^V235fs^ expression led to an increase in cells in G2/M compared with the expression of TREX1^WT^ (Fig. 4F). In addition, within experiments, we were able to exploit the intrinsic mosaic expression of the lentivirus in a single field of view (Fig. 4G). Quantification of EGFP-TREX1 and 53BP1 at an individual cell level showed that EGFP-TREX1^V235fs^ expression levels positively correlated with 53BP1 foci, whilst EGFP-TREX1^WT^ expression shows no correlation (Fig. 4H and I and Supplementary Fig. 10D–G). In summary, the introduction of an RVCL-S-associated *TREX1* mutated allele in human endothelial cells caused accumulation of DNA damage, chromatin bridge formation and cell cycle alterations.

### The cell phenotype caused by the toxic RVCL-S allele can be rescued by targeting exonuclease activity

**Figure 5:**
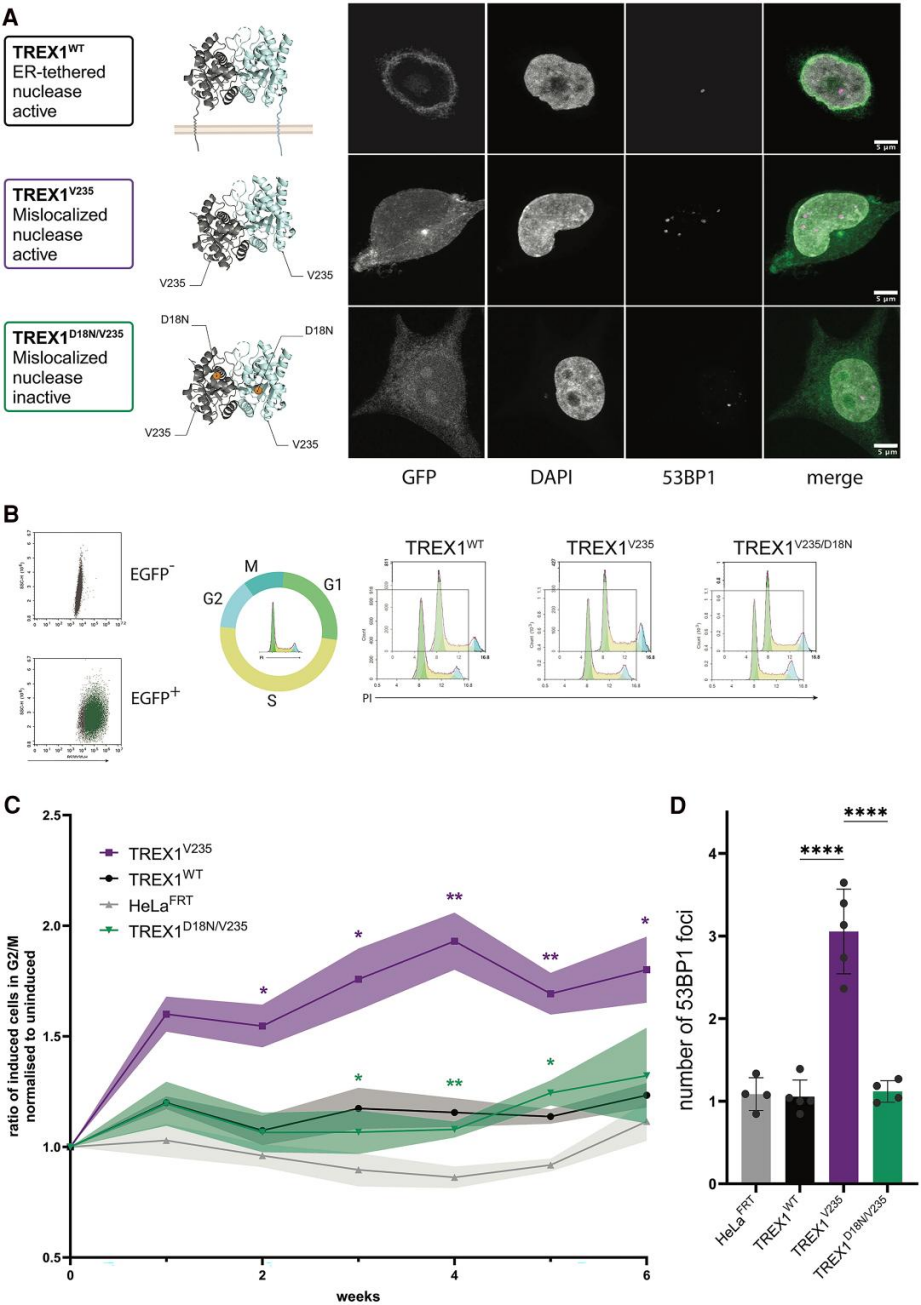
**DNA damage and cell cycle abnormalities in cells expressing TRE­X1^V235fs^, are rescued by inhibition of nuclease activity.** (**A**) Schematic overview of TREX1 dimeric structure generated using Pymol. Created in BioRender. McGlasson, S. (2025) https://BioRender.com/k20e227. The structure of the dimer was extracted from Pymol using RCSB PDB 7TQN. The position of the D18N and V235 mutations were annotated in Pymol. The insoluble C-terminal domains were added manually. Representative images of 53BP1 foci in cells stably expressing EGFP-TREX1 variants, from three independent 6-week experiments. Images were taken on a confocal microscope at ×63. Scale bar = 5 μm. Experiments performed in HeLa cell lines. (**B**) Overview of flow cytometry strategy. EGFP-TREX1 expressing cells were selected via an EGFP gate. EGFP^+^ cells were sorted into cell cycle phases by quantification of propidium iodide (PI) intensity. (**C**) Quantification of proportions of tetracycline-induced cells (EGFP^+^) in G2/M, normalized to uninduced cells (EGFP^−^) at each time point over 6 weeks. Data presented is baseline corrected to uninduced cells from the same cell line at each time point. *P*-values are from a two-way ANOVA with mixed effects, with Tukey’s test for multiple comparisons. *P < 0.05, **P < 0.01; purple stars = EGFP-TREX1^V235fs^ versus EGFP-TREX1^WT^; green stars = EGFP-TREX1^D18N/V235fs^ versus EGFP-TREX1^V235fs^. (**D**) Quantification of 53BP1 foci after 4 weeks of exposure to the EGFP-TREX1 transgene. Data-points are from at least four independent experiments, columns show mean. Error bars are standard error of the mean. *P* < 0.0001 EGFP-TREX1^WT^ versus EGFP-TREX1^V235fs^, *P* < 0.0001 EGFP-TREX1^V235fs^ versus EGFP-TREX1^D18N/V235fs^, one-way ANOVA with Tukey’s correction for multiple comparisons. WT = wild-type.

Our UK Biobank data showed a critical role for intact exonuclease activity in mediating microangiopathic disease in patients with mutations that cause the failure of ER membrane insertion of TREX1. We used our stable human cell lines inducibly expressing *EGFP-TREX1^WT^* or *EGFP-TREX1^V235fs^* to ask whether disease-relevant aspects of the cell phenotype could be rescued by inhibiting the exonuclease activity of the mutant RVCL-S allele. These cells phenocopy the human endothelial cell lines, showing increased DNA damage upon expression of the mutant allele (Fig. 5A).

Therefore, we introduced a well-validated dominant exonuclease-dead mutation (Asp18Asn, D18N) into the *EGFP-TREX1^V235fs^* expression construct to create an additional cell line with a double mutation—*EGFP-TREX1^D18N/V235fs^*. This second mutation completely inhibits exonuclease activity of the untethered TREX1 (Supplementary Fig. 11). The accumulation of cells at the G2/M DNA damage checkpoint was rescued by the double mutant (Fig. 5B and C). Furthermore, accumulation of DNA damage, as measured by 53BP1 foci, was rescued by inhibiting exonuclease activity (Fig. 5D). Therefore, consistent with our findings from the UK Biobank, intact TREX1 exonuclease activity represents a critical therapeutic target in RVCL-S.

## Discussion

RVCL-S is a currently incurable genetic microangiopathic disease. Accurate therapeutic target identification is critical in rare orphan diseases like RVCL-S because of the limited number of clinical trial opportunities. Progress to effective treatments has been hampered by a lack of definitive identification of key disease-relevant pathophysiological steps. Therapeutic approaches to date have been based on proposed mechanisms which suggest a loss of function arising from altered C-terminal interactions with oligosaccharyltransferase.^6^ These studies have implicated activation of the type I interferon response, and have been unsuccessful.

Our study proposes a different mechanism. Our large-scale population-based UK Biobank study, together with *in vitro* analyses of mutated *TREX1* in human cells are consistent with a toxic gain-of-function mechanism based on enzyme misdirection. The ‘toxic product’ is truncated, ER-untethered, enzymatically active TREX1, which would normally be excluded from the nucleus except under specific circumstances.^22-26^ In RVCL-S, active TREX1 exonuclease is found throughout the nuclear compartment. This nuclear TREX1 causes accumulation of DNA damage, resulting in cell cycle dysregulation in endothelial cells. Critically, we have shown that loss of exonuclease activity abrogates pathogenicity of the truncated TREX1. We observe this in our UK Biobank analyses, where more extensive truncations (including and before p.M232) start to lose their dominant pathogenicity once the exonuclease activity of the mutant allele is impacted. Using a complimentary *in vitro* approach, we also observe this phenomenon in our human cellular studies. Specifically, we demonstrate a striking DNA damage phenotype in both HeLa cells and human endothelial cells, resulting from expression of an RVCL-S-causing allele. This phenotype can be fully rescued by targeting exonuclease activity through the introduction of a second, dominant loss-of-function mutation. Both of these approaches suggest that the toxic RVCL-S mutant allele and its exonuclease activity could be targeted therapeutically.

We have shown here how DNA damage can arise in endothelial cells in RVCL-S. Understanding how this DNA damage leads to microvascular disease across multiple organ microvascular beds with consequent multiorgan failure represents a priority for future research. While it is clear from pathological studies that endotheliopathy is a core component, future studies should also consider the role of pericytes and astrocytic endfeet which contribute to the neurovascular/microvascular unit.

We have shown here some of the challenges of using mouse models to study endothelial disease in mice. Although *Trex1* is expressed in endothelial cells, the mutant allele is poorly tolerated in vascular endothelial cells, although we note that our *Tie2-cre* approach has some spatial and temporal limitations given that cre recombinase activity is transiently present in cells of haematopoietic origin. The generation of a mouse model which recapitulates the human microvascular phenotype remains an important challenge for the field.

Our finding that DNA damage may play an important role in the pathogenesis of a monogenic cerebral small vessel disease is of interest. DNA damage is a key driver in both normal ageing and many pathological states.^8^ DNA repair mechanisms deal with DNA damage from many exogenous and endogenous sources throughout a lifetime, however with increasing organism age, these mechanisms become less efficient and DNA damage accumulates.^27,28^ The detrimental effect of DNA damage on vascular endothelial cells has been demonstrated by radiation-induced vascular cognitive impairment which affects up to half of brain tumour survivors.^29^ We believe this is the first small vessel disease where DNA damage has been implicated as a potential primary disease mechanism, and we propose that RVCL-S may constitute a form of premature vascular ageing.

Our findings are particularly timely given the very recent publication of a study with complementary approaches, but a similar broad conclusion that nuclease-mediated DNA damage represents an important disease mechanism in RVCL-S.^8^ These findings of Chauvin *et al.* are predominantly in model organism systems (*Drosophila* and mouse), in contrast to the human population genetic and human endothelial approaches here. However, the human clinical observations in Chauvin *et al.* highlight that previously unrecognized aspects of a DNA damage phenotype may occur in RVCL-S: female patients with RVCL-S exhibit an elevated breast cancer risk, similar to that seen with BRCA1/2 mutations, and RVCL-S patient embryos exhibit genomic instability with chromosomal aneuploidy. This highlights the need to study the pathological consequences of DNA damage in human endothelial cells as exemplified by our experiments, and also highlights the need to study consequences of DNA damage in other cell types. The complementary approaches taken by both papers, arriving at similar findings through differing methodologies, provide synergistic evidence that DNA damage is a key disease-relevant mechanism in RVCL-S.

Our finding that RVCL-S mutations are associated with misdirected nuclease activity and DNA damage in cells, including endothelial cells, has potential implications for cancer surveillance in RVCL-S patients. In addition to elevated breast cancer risk in RVCL-S,^8^ our work raises the possibility that cancer could arise in endothelial cells. To dat,e no such cancers have been reported because cancers of endothelial origin such as angiosarcoma are exceptionally rare. However, our work, together with other recently published findings, heightens the need for vigilance for cancer of breast cancer origin, and also of endothelial cell origin.

Our findings have potential relevance for the development of therapeutic strategies for RVCL-S. To date, clinical trials have primarily focused on targeting immune pathways. Many patients with RVCL-S have received treatment with steroids or other immune treatments,^1^ with limited clinical response. Our findings suggest that an alternative approach would be to consider targeting the exonuclease activity of the mutant allele. Potential therapeutic strategies for further evaluation include the development of TREX1 inhibitors as well as gene silencing of the mutant allele.

In summary, we have shown that the pathogenicity of mono-allelic truncating mutations is critically dependent on intact TREX1 exonuclease function. These RVCL-S-causing mutations cause loss of direct interaction with the ER insertion pathway proteins, with subsequent mislocalization of exonuclease-active TREX1 in the nucleus where it causes DNA damage. Since reducing exonuclease activity in the mutant allele rescues pathogenicity, shown both in our UK Biobank study, and cellular studies, we propose that therapeutic strategies which target this toxic TREX1 product may reduce DNA damage and disease. Such approaches should be prioritized for translational study.

## Supplementary Material

awaf085_Supplementary_Data

## Data Availability

All data will be made publicly available.
