# Supplementary material for: Misdirected yet intact TREX1 exonuclease activity causes human cerebral and systemic small vessel disease
Source: Brain. 2025 Jun 6;148(8):2981–94. doi: 10.1093/brain/awaf085 (PMC12316019; doi:10.1093/brain/awaf085)
Supplement: awaf085_Supplementary_Data [file awaf085_supplementary_data.pdf]

Misdirected yet intact TREX1 exonuclease activity causes human cerebral and systemic small vessel disease

Supplementary Information

Sarah McGlasson[1,2]*, Katy Reid[1,2,♯], Anna Klingseisen[1,2,♯], Bastien Rioux[1,2,♯], Samuel Chauvin[3♯], Cathrine A. Miner[3], Joe Holley[3], Deborah Forbes[1,2], Bethany Geary[4], Jeffrey Kimber[5], Katrina Wood[6], Candice Roufosse[7], Colin Smith[1], David Kavanagh[6], Jonathan Miner[3], David Hunt[1,2]*

Supplementary methods

UK Biobank Participants

The UKB is a population-based cohort of 502000 volunteers recruited between 2006 and 2010 at ages 40-69 years through UK patient registries (response rate: 5.5%)31,32. Self-reported medical conditions at baseline and during follow-up visits (in a subset of participants) were obtained through questionnaires followed by validation interviews conducted by trained staff. Diagnoses are captured both prior and longitudinally after recruitment through linkage with hospital discharge summaries records (primary and secondary diagnoses), national death registries (primary and secondary causes of death), and primary care records (in ~46% of the cohort). Records from linked

general practices can capture diagnoses received through specialist outpatient visits or hospital admissions as reports are generally reported back to general practitioners.

Imaging derived phenotypes

A subset of the cohort underwent brain magnetic resonance imaging (MRI) scanning on 3T Siemens Skyra scanners running VD13A SP4 with a standard Siemens 32-channel radio-frequency receiver head coil33. We used three imaging-derived phenotypes made available to researchers by UKB, which were obtained from validated segmentation algorithms (the Brain Intensity Abnormality Classification Algorithm tool [BIANCA][1] for white matter hyperintensity volume; FMRIB's Automated Segmentation Tool [FAST][2] and SIENAX10[3] for total and hippocampal brain volumes). These volumes were normalized to head size using a standard scaling factor derived from the external surface of the skull (UKB field 25000).

Whole-exome sequencing

Genotypes were defined from whole-exome sequencing data available after sample quality control for 469,229 UKB participants (93.4%). Exome sequences were generated on the Illumina NovaSeq 6000 platform (S2 and S4 flow cells) with a target-enrichment probe kit (IDT xGen® Exome Research Panel v1.0) which enabled deep and uniform coverage of ~39 Mbp. Multi-sample project-level VCF (pVCF) files were made available to researchers after mapping raw sequences with the Original Quality Functionally Equivalent (OQFE) protocol. Variant calls were generated through DeepVariant (v0.10.0), which uses a deep convolutional neural network to determine the most likely genotype at each locus, and GLnexus (v1.2.6), to generate joint genotype pVCF files 37. We applied the following quality control steps to pVCF files as per previous work with UKB exome38:

(i) no withdrawal from the cohort;

(ii) no mismatch between self-reported and genetically determined sex;

(iii) no sex chromosome aneuploidy;

(iv) individual and variant missingness <10%;

(v) Hardy Weinberg equilibrium p-value >10-15;

(vi) at least one sample per site with allele balance threshold >0.15 for SNVs and >0.20 for small indels;

(vii) minimum read coverage depth of seven for SNVs and 10 for indels; and

(viii) sequencing depth ≥10x in 90% of samples (to prevent spurious associations that may result from batch effect).

Variant selection

We considered start loss, frameshift and stop gain variants resulting in a truncating effect from clinical associations and computational predictions (Fig. S4). First, disease-causing truncating TREX1 variants involved in RVCL-S or AGS were systematically retrieved from the National Center for Biotechnology Information (NCBI) ClinVar and the Leiden Open source Variation Database (LOVD) v3.0[4]. Variants were included if they were exclusively reported as pathogenic or likely pathogenic by individual submitters according to the American College of Medical Genetics and Genomics (ACMG) terminology[5], whereas those with a conflicting interpretation of pathogenicity across submitters were included only if none of the submitters had classified them as benign or likely benign (i.e., any conflict was between pathogenic/likely pathogenic and uncertain significance). To capture relevant phenotype associations missing from these databases,

we added variants reported in a comprehensive review of TREX1 genotype-phenotype associations (Rice et al, 2015), a case-control study of individuals with AGS (Rice et al, 2013), and published RVCL-S cases. We assessed the functional impact of disease-causing variants on Ensembl with the Variant Effect Predictor (VEP) and excluded those that did not result in a truncating effect. Second, computationally predicted truncating variants were included to improve statistical power while maintaining a high specificity for pathogenicity. From variants observed in UKB not already included as disease-causing, we added any start loss variants as well as any frameshift or stop gain variants classified as high confidence loss-of-function by the loss-of-function transcript effect estimator (LOFTEE). In addition, to reduce the likelihood of potential misclassification of benign variants as pathogenic, we removed eligible variants fulfilling both of the following criteria:

i) a minor allele frequency ≥0.1% in UKB (whole-exome sequencing), the genome Aggregation Database exome (gnomAD; v2.1), or the 1000 Genomes Project46 (including any subpopulations of these two sequencing databases);

ii) a Combined Annotation-Dependent Depletion (CADD, v1.6) scaled score <10, indicating a lower probability of deleteriousness

Statistical analyses

Association tests for binary outcomes were performed using logistic regressions adjusted for age, sex and the ten first genetic principal components to account for potential population stratification (UKB field 22009). Firth penalization was used to reduce bias in maximum likelihood estimators due to data sparsity. Association tests for brain volumes were performed using linear regressions adjusted for the same covariates, in addition to scanning centre (to control for potential technical

confounding)49. White matter hyperintensity volumes were log-transformed given their right-skewed (log-normal) distribution, and all volumes were z-scored to facilitate interpretation. We defined statistical significance as p-value <0.0014 (i.e., 0.05/34 tests) to account for multiple testing. Analyses were performed on the UKB Research Analysis Platform using JupyterLab (v3.6.1; https://github.com/jupyterlab/jupyterlab) with Hail (v0.2.78; https://github.com/hail-is/hail), Python (v3.9.16; https://github.com/python/cpython) and R (v4.2.0). Logistic regressions were conducted on R using logistf (v1.26.0; https://cran.r-project.org/web/packages/logistf/index.html). Visualization of genomic and phenotype data was conducted on R using trackViewer (https://github.com/jianhong/trackViewer), UpSetR (v1.4.0; https://cran.r-project.org/web/packages/UpSetR/index.html), forestploter (v1.1.1; https://cran.r-project.org/web/packages/forestploter/index.html) and ggplot2 (v.3.4.4; https://cran.r-project.org/web/packages/ggplot2/index.html).

Relative protein quantification by liquid chromatography with tandem mass spectrometry (LC-MS-MS).

Protein digestion

Samples were processed with S-trap micro columns (ProtiFi) using the manufacturer's protocols. Samples were first alkylated with Ttris(2-carboxyethyl)phosphine (TCEP) at 60°C for 30 minutes and then subjected to alkylation in the dark for 30 minutes using Iodoacetamide. Digestion was performed using a Trypsin / Lys-C mix (Pierce) using a 1:20 ratio of enzyme to total sample. The resultant peptide samples were eluted from the S-traps and were vacuum evaporated prior to storage at -20° before mass spectrometry (MS) analysis.

Mass spectrometry

Peptide samples were suspended in 0.1% formic acid in water (v/v) and transferred to vials for MS analysis. The mass spectrometer used was a Thermo Exploris 480 (Thermo) with the liquid chromatography system being a Ultimate 3000 UPLC (Thermo). 1 µg of peptides were injected in 2 µL onto a 5 µm, 100 µm x 2cm nanoViper C18 trap column (Thermo). A 2µm, 75 µm x 50cm C18 reversed phase Easy-spray analytical column was used to resolve peptides over 135 minutes at a flow rate of 300 nl/min. A linear gradient from 3% to 35% was used from water with 0.1% formic acid to acetonitrile with 0.1% formic acid. MS data was acquired in data-independent mode using a 45 variable m/z window method.

Data analysis

Resultant mass spectrometry .raw files were processed using DIA-NN (version 1.8.1 doi: 10.1038/s41592-019-0638-x) with the library-free search option enabled. For the reference database a human uniprot FASTA (downloaded on 16/05/2021) was used. The default settings were used except for Heuristic protein inference. Protein inference was set to "Protein names (from FASTA)" and the neural network classifier was used in double-pass mode. The protein abundance matrix was then processed using R (version 4.1.3). Abundance values were subjected to quantile normalisation using functions from the PreProcessCore package. Missing values were imputed using the ImpSeqRob function as part of the rrcovNA package. Statistical comparisons were performed using the Limma package using the "eBayes" method with FDR correction for multiple testing.

Endothelial tube forming assay

Tube formation assays were performed as directed by Corning https://www.corning.com/catalog/cls/documents/protocols/protocol_DL_030_Endothelial_Cell_Tube_Formation_Assay.pdf). Briefly, cell culture plates were coated with Matrigel (10 mg/ml, Corning Cat. No. 354234) making sure to keep matrigel, pipettes tips and plates ice cold to prevent premature gelling. Plates were incubated in the Incucyte live cell analysis system (Sartorius) for 30-60 minutes. Cells were seeded at varying cell densities onto the matrigel and incubated with imaging at regular time points. Images were analysed in the Incucyte software.

hBEC RT-PCR

For RT-PCR, total mRNA was isolated from induced pluripotent stem cells (iPSC, WT cells, ED027, kind gift from Dr. Katherine Bowles), from early time-point differentiated iPSC-derived endothelial cells (protocol after Bertucci et al., 2023[6]), human umbilical vein endothelial cells (HUVEC, Lonza #C2519A), Hbec-5i (ATCC CRL-3246) and Hela cells. RNA was isolated using the QIAwave DNA/RNA Mini Kit (Quiagen, Cat. No 80504) and cDNA prepared using the High capacity cDNA reverse transcription Kit (Applied Biosystems, Thermo Fisher Scientific Cat. No 4368814). 1 µl template cDNA was used for PCR amplification of endothelial markers.

List of primers used

Gene	Synthetic oligonucleotide	Expected product
ACTB	Fw 5’-GAGAAAATCTGGCACCACACC-3’<br>Rv 5’-CGACGTAGCACAGCTTCTC-3’	412bp

OCT4	Fw 5’-CGTAAGCAGAAGAGGATCACC-3’<br>Rv 5’-GCTTCCTCCACCCACTTCTGC-3’	179bp
SOX2	Fw 5’-GCAGCTACAGCATGATGCAGG-3’<br>Rv 5’-AGCTGGTCATGGAGTTGTACTGC-3’	134bp
KDR /VEGFR2	Fw 5’-TGCAAGGACCAAGGAGACTCTGT-3’<br>Rv 5’-TAGGATGATGACAAGAAGTAGCC-3’	458bp
VEC /VE Cadherin	Fw 5’-CAGCCCAAAGTGTGTGAGAA-3’<br>Rv 5’-TGTGATGTTGGCCGTGTTAT-3’	162bp
PECAM 1 /CD31	Fw 5’-AGGTCAGCAGCATCGTGGTCAACAT-3’<br>Rv 5’-GTGGGGGTTGTCTTTGAATACCGCAG-3’	187bp

Systemic literature review of RVCL-S endothelial pathology

We systematically reviewed the literature for evidence of endotheliopathy within biopsy specimens. The literature was searched for “RVCL-S” and “retinal vasculopathy with cerebral leukodystrophy/leukoencephalopathy and systemic manifestations) and all papers identified (**Fig. 3C**). The identification of relevant papers was undertaken by two authors who reviewed all papers and identified those where there were sufficient pathological details to support future review. This identified 12 papers where sufficient pathological material was available. Pathological images and text relating to pathology was then reviewed by an experienced pathologist (CS). A paper was considered to have positive evidence of endotheliopathy if there was evidence of this on pathology images, or reference to specific endotheliopathic features of biopsy material in the text.

TREX1-dsRED mouse flow cytometry

The TREX1-dsRed flow cytometry data were generated in the Penn Cytomics and Cell Sorting Shared Resource Laboratory at the University of Pennsylvania and is partially supported by the

Abramson Cancer Center NCI Grant (P30 016520). The research identifier number is RRID:SCR_022376.

Protocols for mouse studies were approved by the Institutional Animal Care and Use Committee (IACUC) at Penn.

Human TREX1 mice have a CAG promoter, a transcriptional stop sequence flanked by loxP sites, and N-terminal HA-tagged WT human TREX1 or human TREX V235Gfs in the ROSA26 locus. To get expression in endothelial cells, floxed-STOP human TREX1 mice were crossed to Tie2-Cre animals (Jax 008863). Mice were housed in pathogen-free facilities at the University of Pennsylvania Perelman School of Medicine and fed standard diet and water *ad libitum*.

TREX1-dsRed reporter mice were previously described[7]. Briefly, these mice express wild-type mouse TREX1 as well as the dsRed reporter at the endogenous locus, under control of the endogenous promoter. Mice were housed in pathogen-free facilities at Penn and fed a standard diet ad libitum. Experiments were performed using mice of both sexes at ages 5-8 weeks.

To analyze TREX1 expression in splenic endothelial cells, spleens were digested as previously described[8]. Each spleen was injected with 2 mL of a digestion buffer containing 2 mg/mL of collagenase D (Sigma; 11088866001), 0.8 mg/mL of dispase II (Sigma; D4693), and 0.1 mg/mL of DNase I (Sigma; DN25-10MG) in RPMI-1640 (Thermo Fisher; 11875093) with 2% (v/v) heat inactivated fetal bovine serum (FBS, Omega Scientific; FB-01). The spleens were digested for 30 minutes at 37˚C before mixing with a 1 mL pipette until no pieces of tissue remained visible. The cell suspension was washed in PBS with 2% FBS (FACS buffer) before lysing red blood cells with ACK lysis buffer (Gibco; A1049201) for 3 minutes. To assess viability, cell suspensions were stained with Zombie NIR fixable viability dye (BioLegend; 423106) in PBS

for 15 minutes on ice. Cells were then stained for CD45 (AF700, BioLegend; 103127), Ter119 (AF700, BioLegend; 116220), CD31 (BV421, BioLegend; 102423), and podoplanin (PerCP/Cy5.5, BioLegend; 127421) in FACS buffer for 30 minutes on ice. Fc-mediated interactions were blocked by incubating cell suspensions with purified rat anti-mouse CD16/32 (BD Biosciences; 553142) during cell surface staining. Cells were analyzed on an LSR II (BD Biosciences) and data analysis was conducted in FlowJo v10 software (FlowJo LLC).

Analysis of TREX1 expression in human brain via single nuclei RNA sequencing

Raw data was extracted from a publically available dataset[9] . STAR version 2.7.11b was used to prepare a Homo Sapiens (primary build GRCh38 from Ensembl release 84) genome suitable for use by STAR using the gtf and fasta files provided by 10x. The fastq data for each sample was then processed with the following parameters: runThreadN 8, soloType Droplet, soloCBwhitelist 3M-february-2018.txt, soloCBmatchWLtype 1MM_multi_Nbase_pseudocounts, soloUMIfiltering MultiGeneUMI_CR, soloUMIdedup 1MM_CR, soloCellFilter EmptyDrops_CR, soloFeatures GeneFull, soloMultiMappers EM. readFilesCommand "zcat -f". For each sample a Seurat object was created with the 'parameters min.cells=1' and 'min.features=10'. The Seurat object was filtered to remove those samples not present in the Seurat object from the original publication[9]. The meta data from the Seurat object was transfered to the new Seurat object. Each Seurat object was saved as a separate RDS file. This was done using Seurat Version 4 with R version 4.0.0. The remaining steps were run using R 4.3.0 version and Seurat version 5.0.1. Each per sample Seurat object was merged to create a single Seurat object. The merged object was normalised, variable features selected and data scaled and PCA reduction

performed, all using default parameters. Data was integerated using the 'integrated.cca' reduction. Umap analysis was performed using the parameters 'dims = 1:25, reduction = "integrated.cca"'. As described in the original publication [9] endothelial cells and pericytes can share the expression of genes that mark blood vessel types. We, therefore, used the scSorter[10] with weighted markers for blood vessel type annotation as described in Quick et al.[11] We then manually annotated pericytes and endothelial cells based on expression of PDGFRB and VEGFC.

Analysis of *TREX1* expression in human and mouse brain via bulk RNA sequencing

Normalised data was extracted from a publically available database (Barres lab BrainRNAseq) [12,13]. For simplicity the human data 'feotal astrocytes' and 'adult astrocyte' were combined into a single 'Astrocyte' group. For simplicity the mouse data 'myelinating oligodendrocytes' and 'newly formed oligodendrocytes' were combined into a single 'oligodendrocyte' group.

Western blot analysis

Whole cell extracts were obtained by lysis in RIPA buffer with complete mini protease inhibitor cocktail and protein concentration was quantified by BCA assay before resolution by SDS-PAGE following standard procedures. Samples were run on 12% acrylamide SDS-PAGE or NuPage minigels at 200V until the blue loading dye ran to the bottom of the gel, and transferred to nitrocellulose or PVDF membranes at 100 V in a cold room for 1 hour.

Membranes were blocked in Intercept® (TBS) Blocking Buffer (Licor) for 1 hour at room temperature on a roller. Primary incubations were performed in blocking buffer with tween 20 (0.2%) overnight at 4°C on a roller. The membrane was washed 3 times with TBS-Tween 20

(0.1%), and incubated with secondary antibody in block with tween 20(0.2%). The wash step was repeated and the membrane was imaged on the Licor Odyssey DLx Imager. EGFP-TREX1 was detected using anti-GFP antibody (JL-8, Living Colors® A.v. mAb) and IRDye® Secondary Antibodies (Licor) were used.

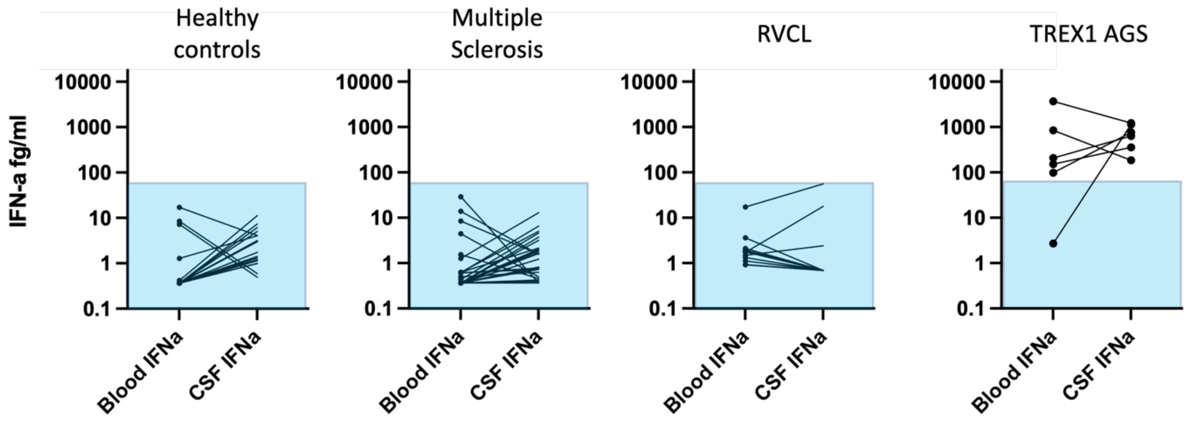

Supplementary Figure 1 Ultrasensitive IFN-alpha biomarkers show that RVCL is not an interferonopathy. Systematic analysis of paired serum-CSF samples, including healthy and disease controls (multiple sclerosis, a non-interferonopathic neurological disease), together with paired analyses of RVCL and TREX1-associated Aicardi-Goutières' Syndrome. This is a meta-analysis of previously published data from our lab and others [14 15]

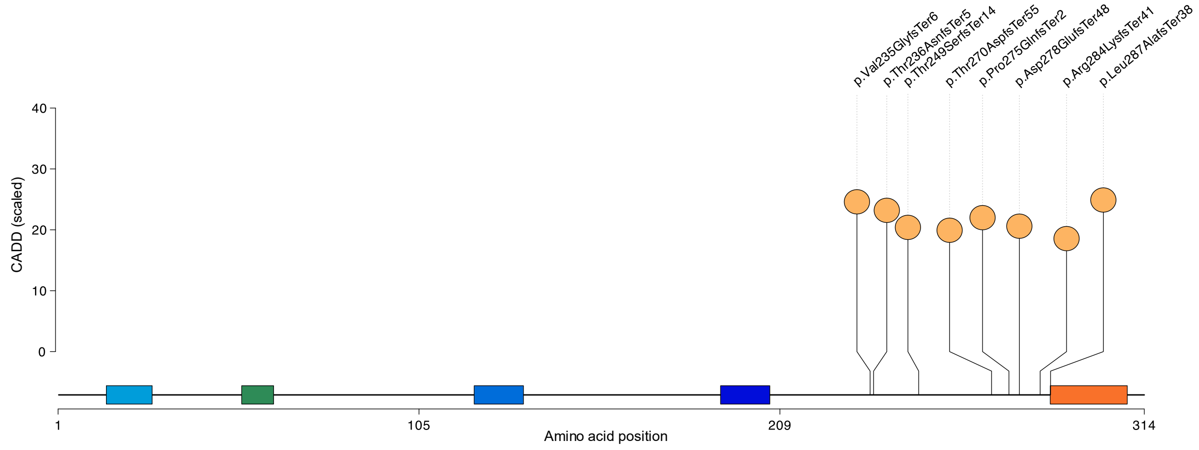

Supplementary Figure 2 All published RVCL-causing mutations in *TREX1*. Domains: ExoI=light blue, ExoII=mid-blue, ExoIII=dark blue, PPII=green, ER TMD=orange. Protein position is annotated from NM_033629.6 (*TREX1*). This

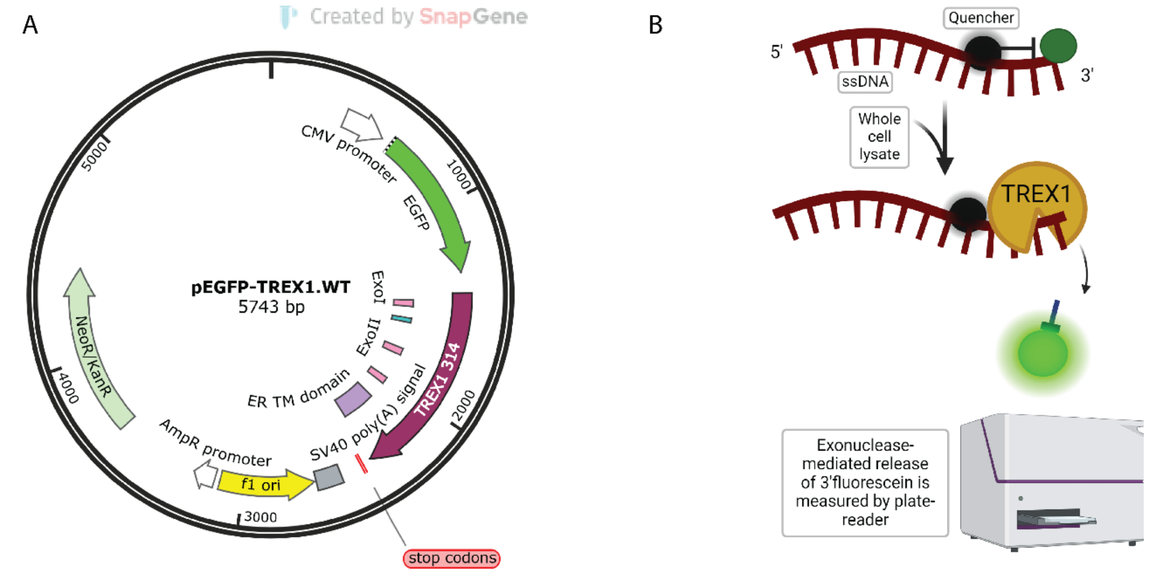

Supplementary Figure 3 Functional assay of *TREX1* variants by transient expression of an EGFP-TREX1 fusion protein for imaging and measurement of single stranded 3'-5' exonuclease activity.

A) An EGFP-C2 plasmid was adapted for gateway cloning and the coding sequence of *TREX1* (CCDS2769.1) was cloned into the plasmid so that the EGFP was an N-terminal fusion. This plasmid was transiently expressed in *Trex1*$^{-/-}$ MEFs for 24 hours before analysis via microscopy and total protein lysate extraction. Created by Snapgene

B) A fluorescent plate-based assay was established and optimised for high throughput analysis of TREX1 variant function. A single-stranded DNA oligo with 5'fluorescein and an internal quencher (Dabcyl) was incubated with whole cell lysate. 3'-5' exonuclease activity was assayed by measuring fluorescence released over time. Created with BioRender.com

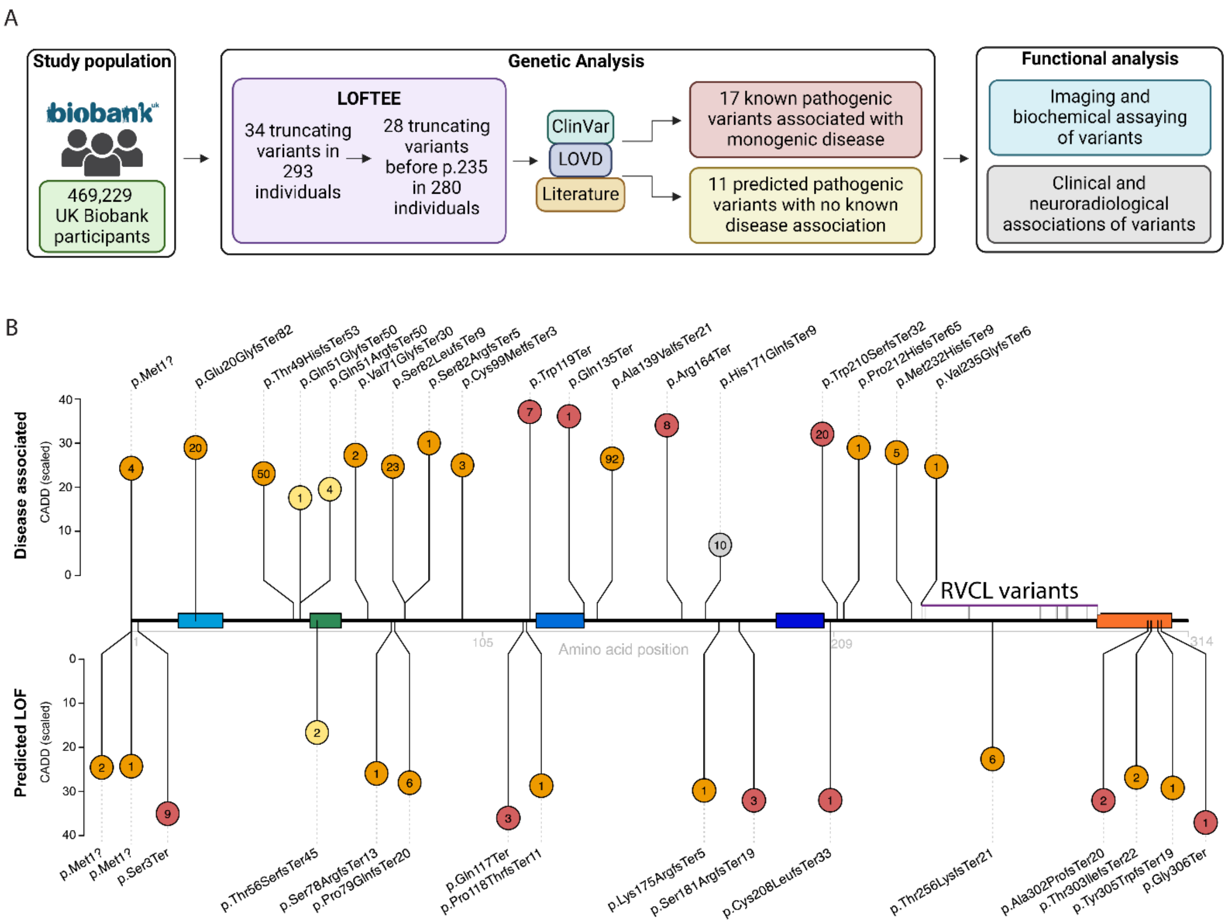
A
Study population
biobank
469,229 UK Biobank participants
Genetic Analysis
LOFTEE
34 truncating variants in 293 individuals
28 truncating variants before p.235 in 280 individuals
ClinVar
LOVD
Literature
17 known pathogenic variants associated with monogenic disease
11 predicted pathogenic variants with no known disease association
Functional analysis
Imaging and biochemical assaying of variants
Clinical and neuroradiological associations of variants
B
Disease associated
CADD (scaled)
Predicted LOF
CADD (scaled)
Amino acid position
RVCL variants
p.Met1?
p.Glu20GlyfsTer82
p.Thr49HisfsTer53
p.Gln51GlyfsTer50
p.Gln51ArgfsTer50
p.Val71GlyfsTer30
p.Ser82LeufsTer9
p.Ser82ArgfsTer5
p.Cys99MetfsTer3
p.Trp119Ter
p.Gln135Ter
p.Ala139ValfsTer21
p.Arg164Ter
p.His171GlnfsTer9
p.Trp210SerfsTer32
p.Pro212HisfsTer65
p.Met232HisfsTer9
p.Val235GlyfsTer6
p.Met1?
p.Met1?
p.Ser3Ter
p.Thr56SerfsTer45
p.Ser78ArgfsTer13
p.Pro79GlnfsTer20
p.Gln117Ter
p.Pro118ThrfsTer11
p.Lys175ArgfsTer5
p.Ser181ArgfsTer19
p.Cys208LeufsTer33
p.Thr256LysfsTer21
p.Ala302ProfsTer20
p.Thr303IlefsTer22
p.Tyr305TrpfsTer19
p.Gly306Ter

Supplementary Figure 4 An atlas of truncating *TREX1* variants identified in UK Biobank

A) Schematic overview of selection, filtering and annotation of truncating *TREX1* variants in UK Biobank. We searched UK Biobank for any truncating *TREX1* variants and filtered these further into those variants that cause more extensive truncation than RVCL-causing mutations (i.e. before V235). Using LOVD, ClinVar and published case reports we filtered all of the truncating variants into known disease associated variants and predicted loss-of-function variants. All variants were functionally assessed at a phenotype level and a subset at cellular level. Created with BioRender.com

B) Lollipop plot of all truncating *TREX1* variants present in the UK Biobank. Above x-axis is known disease-associated mutations, below x-axis is predicted loss-of-function variants. Each node represents a variant with its carrier count in the UK Biobank and its CADD score (scaled colour and Y-axis).

Phenotype	Non-carriers, n (%)	Carriers, n (%)	aOR (95% CI)	p-value
Neurological and cognitive				
Dementia	9,333 (2.0)	5 (1.8)	0.88 (0.33, 1.88)	0.765
Alzheimer's disease	4,139 (0.9)	2 (0.7)	0.89 (0.19, 2.54)	0.850
Vascular dementia	2,075 (0.4)	2 (0.7)	1.84 (0.38, 5.26)	0.387
Stroke	19,607 (4.2)	6 (2.1)	0.51 (0.21, 1.02)	0.057
Ischemic stroke	16,647 (3.5)	5 (1.8)	0.51 (0.19, 1.07)	0.080
Encephalitis	991 (0.2)	1 (0.4)	2.59 (0.30, 9.26)	0.316
Epilepsy	8,363 (1.8)	3 (1.1)	0.70 (0.20, 1.74)	0.491
Migraine	25,663 (5.5)	10 (3.5)	0.69 (0.35, 1.21)	0.211
Brain neoplasm	943 (0.2)	1 (0.4)	2.72 (0.31, 9.74)	0.295
Multiple sclerosis and others	2,871 (0.6)	1 (0.4)	0.92 (0.11, 3.31)	0.922
Ocular				
Glaucoma	19,583 (4.2)	14 (5.0)	1.15 (0.64, 1.89)	0.614
Retinopathy	21,880 (4.7)	14 (5.0)	0.99 (0.55, 1.63)	0.971
Visual impairment	4,176 (0.9)	3 (1.1)	1.31 (0.37, 3.23)	0.635
Other systemic				
Anaemia	34,274 (7.3)	11 (3.9)	0.49 (0.26, 0.85)	0.009
Chronic kidney disease	30,618 (6.5)	14 (5.0)	0.71 (0.40, 1.17)	0.191
Hypertension	189,438 (40.4)	116 (41.1)	0.96 (0.74, 1.23)	0.722
Hypothyroidism	38,190 (8.1)	18 (6.4)	0.73 (0.44, 1.15)	0.185
Liver disease	17,575 (3.7)	9 (3.2)	0.87 (0.42, 1.56)	0.658
Raynaud	5,729 (1.2)	4 (1.4)	1.31 (0.44, 2.96)	0.585
Spontaneous abortion	5,417 (2.1)	4 (2.5)	1.34 (0.44, 3.07)	0.561
Psychiatric				
Anxiety	41,731 (8.9)	19 (6.7)	0.78 (0.48, 1.20)	0.275
Depression	58,646 (12.5)	32 (11.3)	0.94 (0.64, 1.34)	0.743
Psychosis	1,430 (0.3)	0 (0.0)	0.558 (0.004, 3.791)	0.648
Systemic inflammation				
Lupus/Sjogren's	2,642 (0.6)	1 (0.4)	0.80 (0.09, 2.88)	0.782
Vasculitis	2,164 (0.5)	2 (0.7)	1.83 (0.38, 5.17)	0.389
Any	293,752 (62.6)	173 (61.3)	0.90 (0.70, 1.15)	0.384

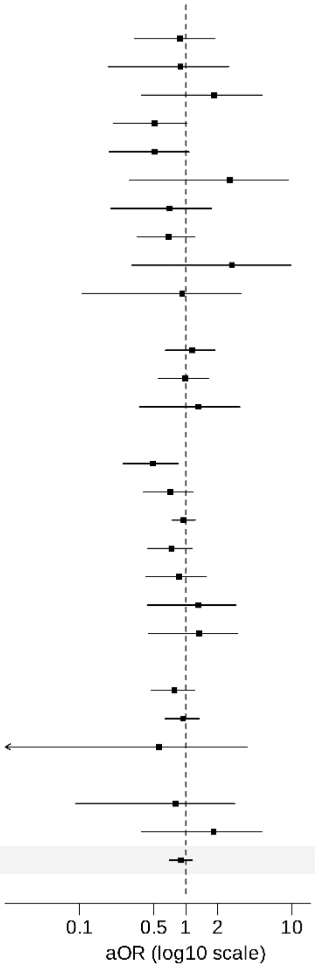

Supplementary Figure 5. Full breakdown of clinical phenotype associations with truncating TREX1 variants in the UK Biobank.

Forest plot showing full clinical phenotype associations of truncating *TREX1* variants in the UK Biobank. The frequency of 30 RVCL-associated outcomes, summarized into 5 categories, were tested using logistic regression. aOR, adjusted odds ratio; CI, confidence interval.

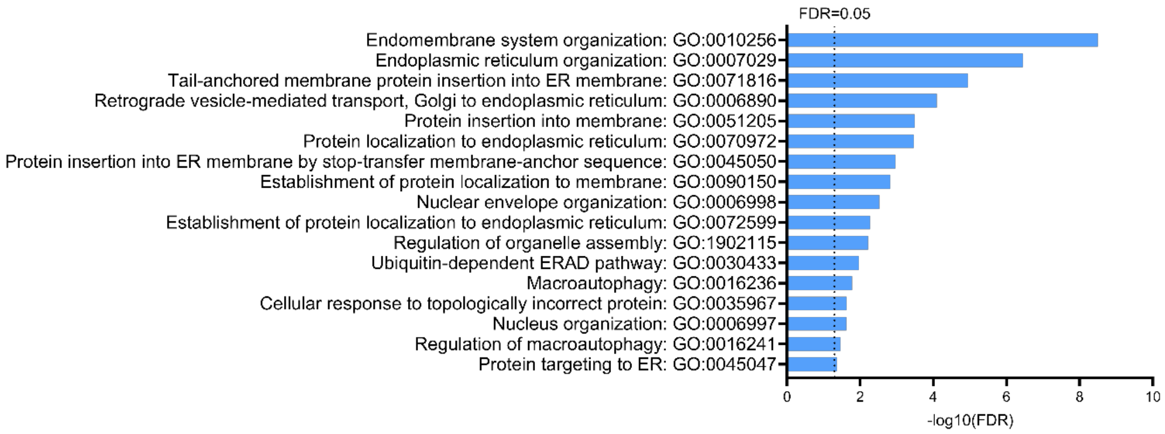

Supplementary Figure 6 Analysis of GO process terms of proteins that are significantly lost by TREX1^{V235fs} vs TREX1^{WT}

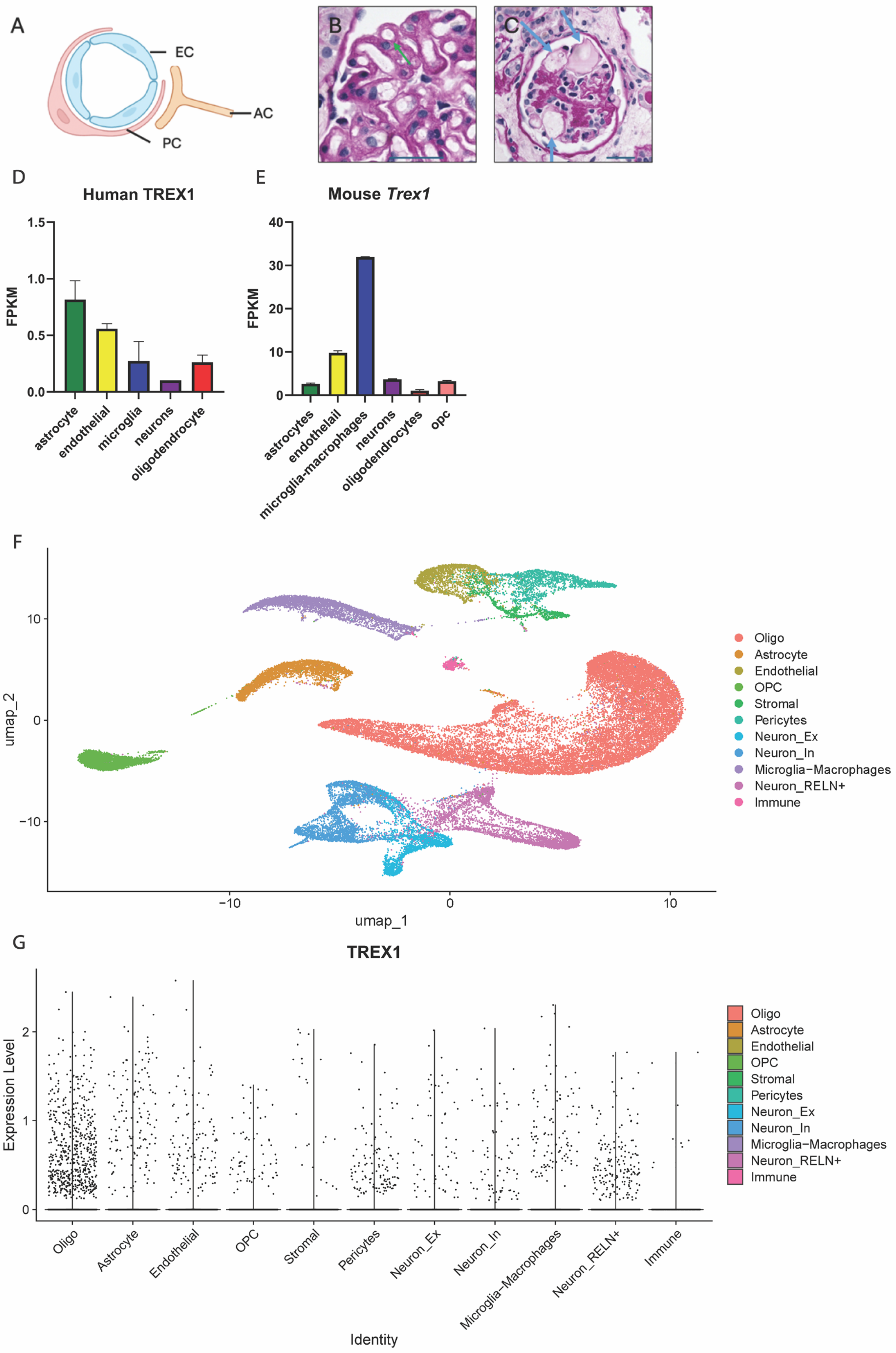
A
EC
AC
PC
B
C
D
Human TREX1
FPKM
astrocyte
endothelial
microglia
neurons
oligodendrocyte
E
Mouse *Trex1*
FPKM
astrocytes
endothelail
microglia-macrophages
neurons
oligodendrocytes
opc
F
umap_2
umap_1
Oligo
Astrocyte
Endothelial
OPC
Stromal
Pericytes
Neuron_Ex
Neuron_In
Microglia−Macrophages
Neuron_RELN+
Immune
G
TREX1
Expression Level
Identity

Supplementary Figure 7. The neurovascular unit and *TREX1* expression in the brain

A) Schematic of the human neurovascular unit showing endothelial cells (EC), pericytes (PC) and astrocytic endfeet (AC).

B) Evidence of basement membrane double contouring, suggestive of endothelial injury (green arrows) seen in patient renal biopsy (Periodic Acid-Schiff x 400, same biopsy as figure 3) Scale bar = 100um

C) In this renal biopsy evidence of microaneurysms are found (blue arrows), which have been associated with pericyte disease. Scale bar = 100um

D, E) Expression of human *TREX1* and mouse *Trex1* in brain cell types extracted from a bulk RNA sequencing database (Barres lab BrainRNAseq[12,13])

F) Single nuclear RNA sequencing (snRNAseq) analysis was performed on normal human brain tissue[9]. In order to test *TREX1* expression at the single cell level, we realigned raw snRNAseq reads using STARsolo[16], since data that has been analysed using Cell Ranger (i.e. 10X data) will exclude *TREX1* based on its complete overlap with the 3'UTR of *ATRIP*. Uniform Manifold Approximation and Projection (UMAP) plots demonstrate that the major cell types of the human brain could be identified as distinct clusters.

G) *TREX1* expression could be identified, albeit at low levels, in all major cell types of the human brain.

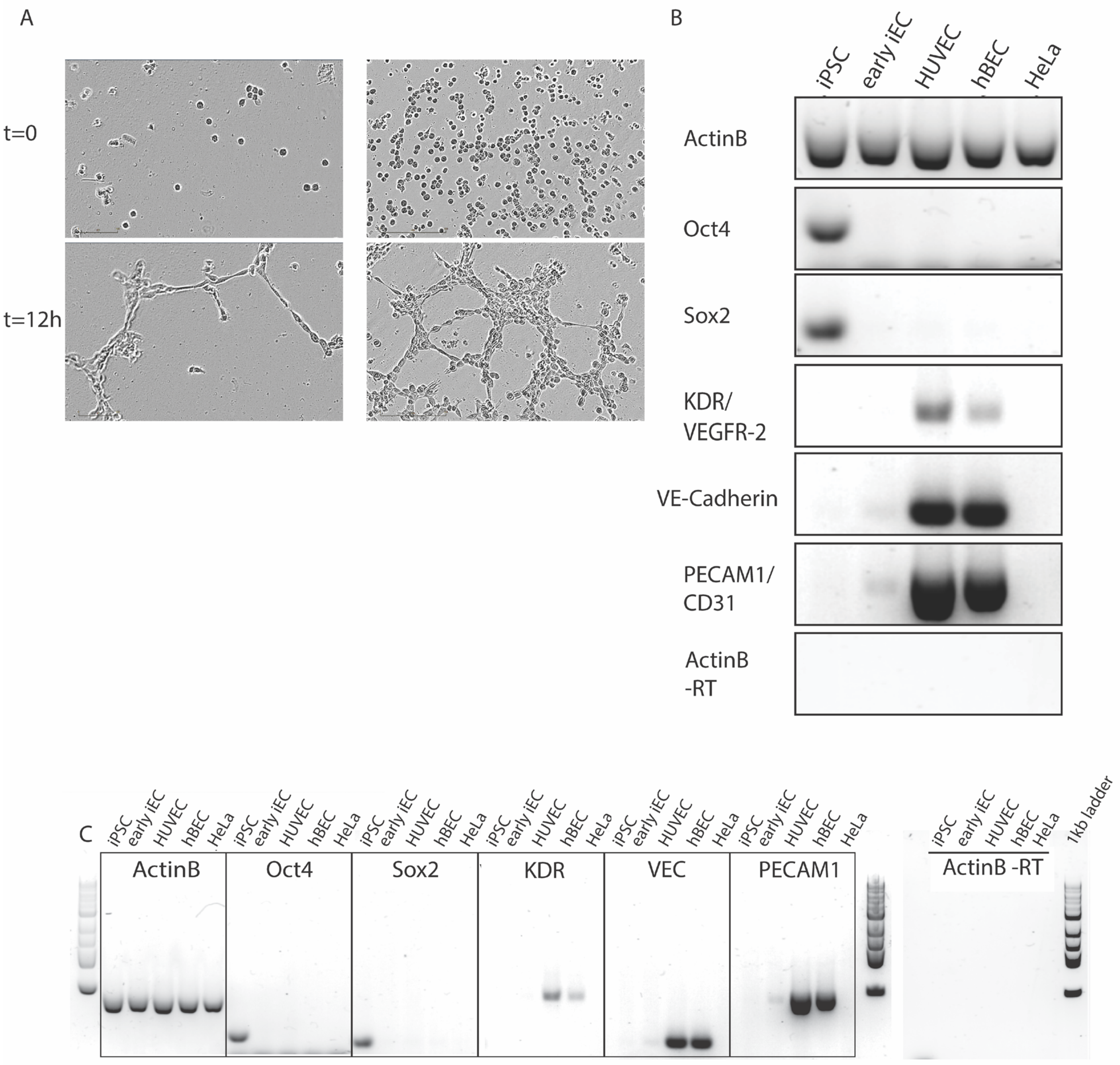

A
t=0
t=12h
B
iPSC
early iEC
HUVEC
hBEC
HeLa
ActinB
Oct4
Sox2
KDR/
VEGFR-2
VE-Cadherin
PECAM1/
CD31
ActinB
-RT
C
ActinB
Oct4
Sox2
KDR
VEC
PECAM1
ActinB -RT
1kb ladder

Supplementary figure 8. Characterisation of hBEC cells

A) Formation of tube-like structures by hBECs after 12 hours of culture on matrigel. Scale bar = 200 μm. 2 representative images from independent experiments

B) RT-PCR of endothelial marker genes in induced pluripotent stem cells (iPSC), early iPSC-derived endothelial cells (early iEC), human umbilical vein endothelial cells (HUVEC), hBEC and HeLa cells. ActinB is a loading control.

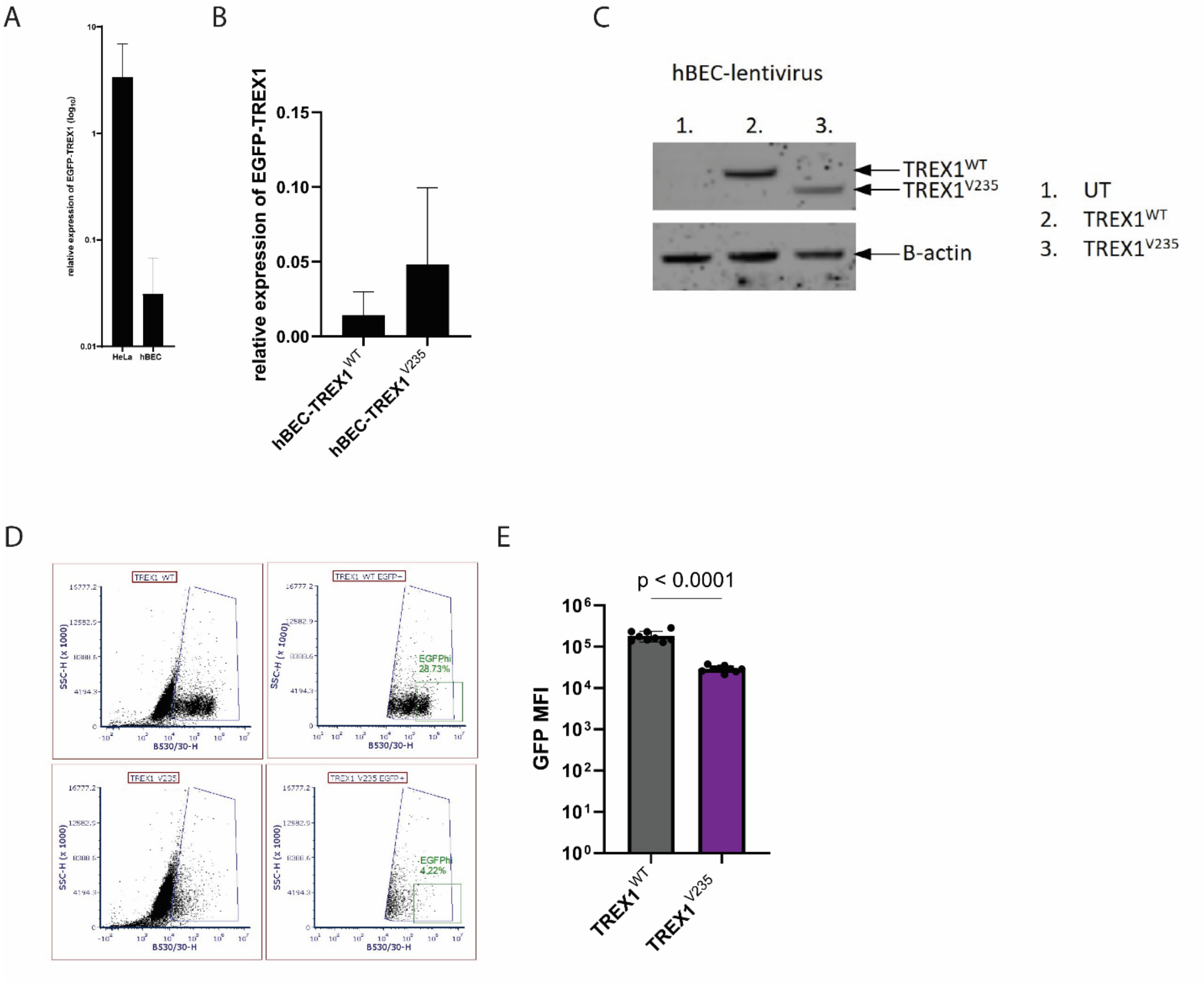

Supplementary figure 9. Validation of TREX1 transgenic expression in hBEC and HeLa cells.

(A) Transgenic expression in induced HeLa cells vs lentivirally transfected hBEC cells

(B) Quantification of expression of $TREX1^{WT}$ vs $TREX1^{V235}$ in lentivirally transfected hBEC cells quantified by qPCR. Normalised to housekeeping gene GAPDH. N=2 HeLa, n=4 hBEC

(C) Expression of $TREX1^{WT}$ vs $TREX1^{V235}$ protein in lentivirally transfected cells tested by western blotting against GFP. Representative of 4 experiments

(D) Representative flow cytometry plot of HeLa cells expressing EGFP-TREX1^{WT} or EGFP-TREX1^{V235} . Representative of 9 time points from 3 independent experiments

(E) Quantification of median fluorescence intensity (MFI) of GFP in HeLa cells expressing EGFP-TREX1^{WT} or EGFP-TREX1^{V235}. Data points show 9 time points from 3 independent experiments, column shows mean, error bars show SEM, unpaired t-test. Y axis is log transformed.

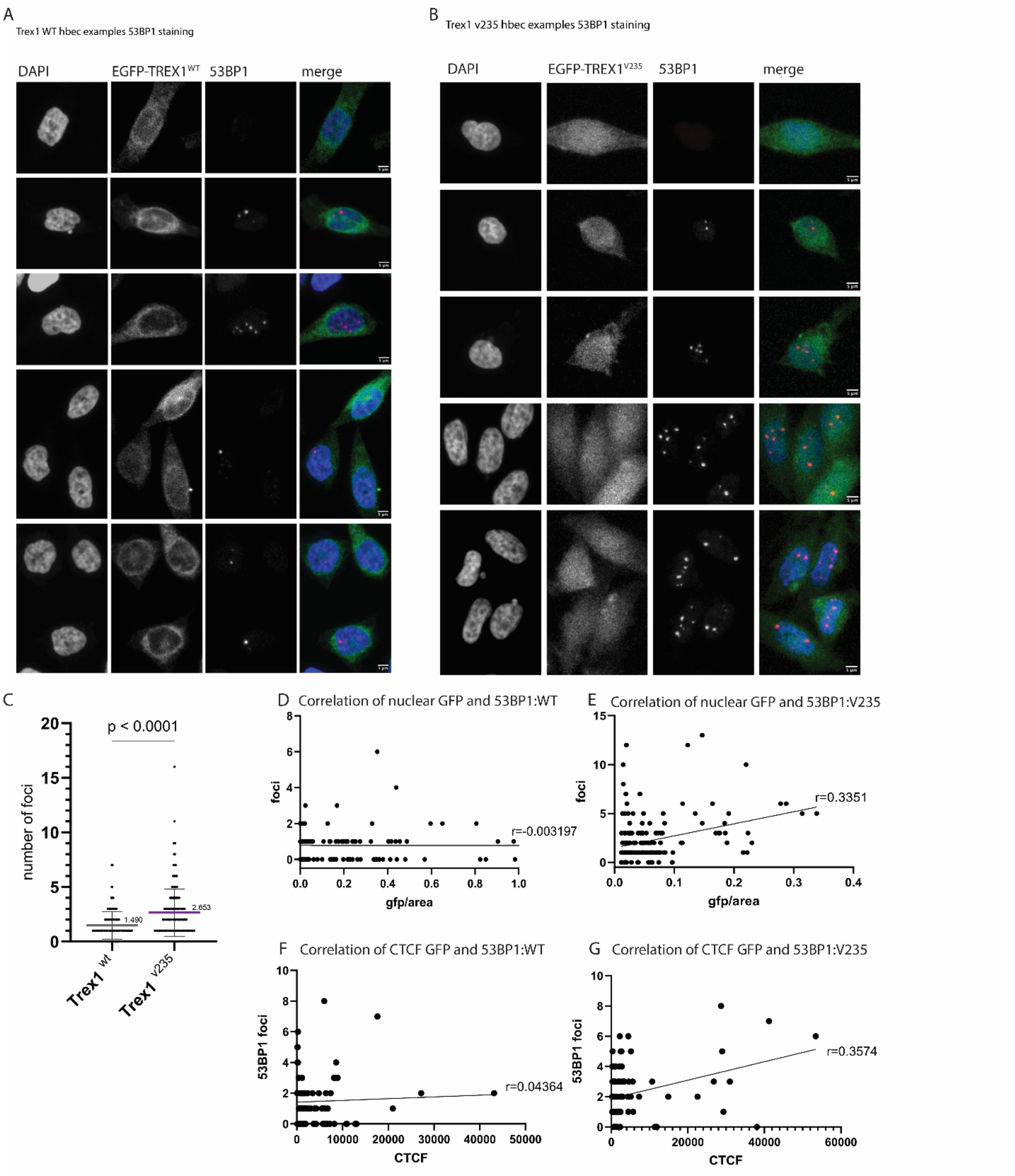
A
Trex1 WT hbec examples 53BP1 staining
DAPI
EGFP-TREX1WT
53BP1
merge
B
Trex1 v235 hbec examples 53BP1 staining
DAPI
EGFP-TREX1V235
53BP1
merge
C
p < 0.0001
number of foci
1.490
2.653
Trex1 wt
Trex1 v235
D Correlation of nuclear GFP and 53BP1:WT
foci
gfp/area
r=-0.003197
E Correlation of nuclear GFP and 53BP1:V235
foci
gfp/area
r=0.3351
F Correlation of CTCF GFP and 53BP1:WT
53BP1 foci
CTCF
r=0.04364
G Correlation of CTCF GFP and 53BP1:V235
53BP1 foci
CTCF
r=0.3574

Supplementary Figure 10. **Further characterization of DNA damage in hBECs at single cell resolution**

(A, B) Example images of different foci numbers across multiple (n=3 WT, n=4 V235) independent experiments.

(C) Distribution of foci counts across independent experiments (n=3 WT, n=4 V235)

(D, E) Correlation of 53BP1 foci counts with nuclear GFP across independent experiments (n=3 WT, n=4 V235). Correlation calculated by Pearson correlation coefficient (r). Line fit with simple linear regression.

(F, G) Correlation of 53BP1 foci counts with corrected total cell fluorescence (CTCF) of GFP (n=2 per group). Correlation calculated by Pearson correlation coefficient (r). Line fit with simple linear regression.

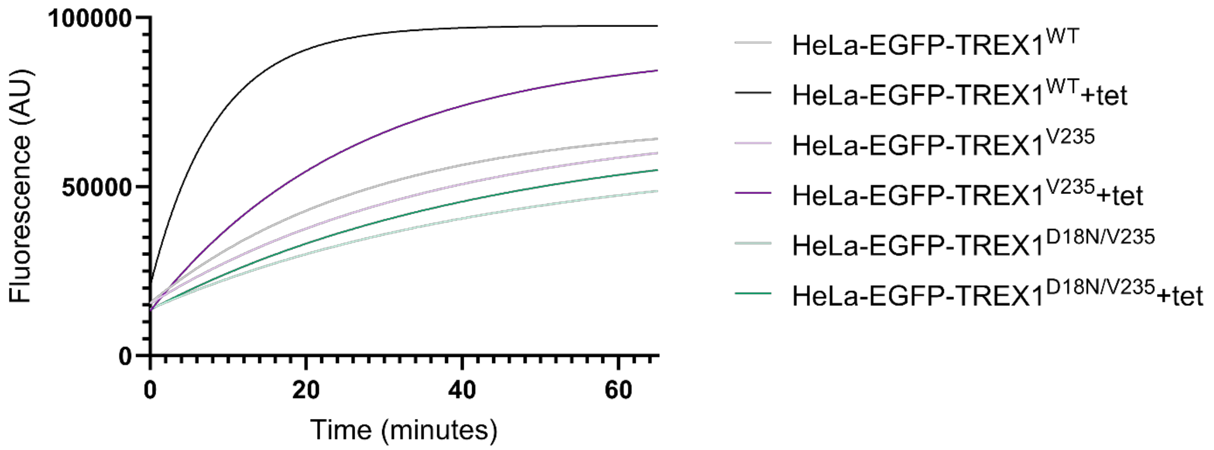

Supplementary Figure 11. Validation of 3’-5’ exonuclease activity in stable, inducible HeLa cell lines.

Total protein lysate was extracted from UT or tetracycline treated HeLa lines to assay 3’-5’ exonuclease activity over time. Data presented is from 3 independent experiments, with non-linear curve fit.

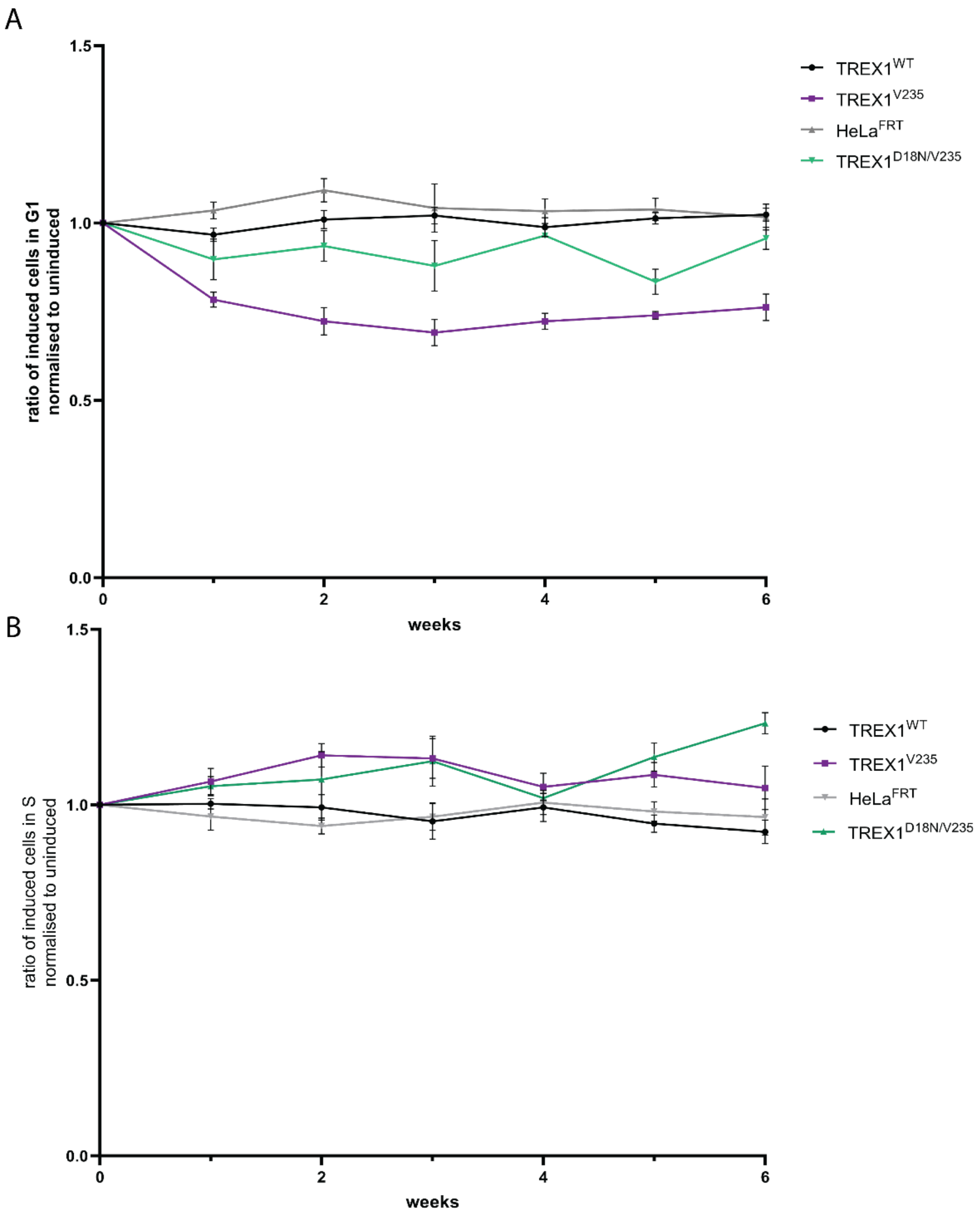

Supplementary Figure 12. G1 and S phase are unaffected in stable HeLa cells.

Supplementary Table 1. All truncating TREX1 variants identified in UK Biobank.

34 distinct TREX1truncating variants were identified in 293/469229 UK Biobank participants

HGVSc; HGVSp	**Source**	**CADD (scaled)**	**Genotypes in the UKB**		**Known phenotype association**	
			**Het**	**Hom**		
c.1A>G; p.Met1?	ClinVar	24.2	4	0	AGS	
c.2T>C; p.Met1?	Predicted	24.5	2	0		
c.3G>C; p.Met1?	Predicted	24.3	1	0		
c.8C>A; p.Ser3Ter	Predicted	35.0	9	0		
c.58dup; p.Glu20GlyfsTer82	ClinVar	28.9	20	0	AGS	[17]
c.144dup; p.Thr49HisfsTer53	ClinVar	23.0	50	0	AGS	
c.150_151del; p.Gln51GlyfsTer 50	ClinVar	17.6	1	0	AGS	[18]
c.152_153del; p.Gln51ArgfsTer 50	ClinVar	19.5	4	0	AGS	[19]
c.167_168del; p.Thr56SerfsTer4 5	Predicted	16.6	2	0		

c.212_213del; p.Val71GlyfsTer30	ClinVar	27.2	2	0	AGS	
c.226_233dup; p.Ser78ArgfsTer13	Predicted	25.9	1	0		
c.236_243dup; p.Ser82LeufsTer9	ClinVar	24.6	23	0	AGS	
c.236_243del; p.Pro79GlnfsTer20	Predicted	28.0	6	0		
c.243_246del; p.Ser82ArgfsTer5	ClinVar	29.9	1	0	AGS	
c.294dup; p.Cys99MetfsTer3	LOVD	24.9	3	0	AGS	[20]
c.348_351dup; p.Pro118ThrfsTer11	Predicted	28.7	1	0		
c.349C>T; p.Gln117Ter	Predicted	36.0	3	0		
c.357G>A; p.Trp119Ter	ClinVar	37.0	7	0	AGS	
c.403C>T; p.Gln135Ter	ClinVar	36.0	1	0	AGS	
c.416del; p.Ala139ValfsTer21	ClinVar	26.4	92	0	AGS	

c.490C>T; p.Arg164Ter	ClinVar	34.0	8	0	AGS	
c.513del; p.His171GlnfsTer9	ClinVar	6.9	10	0	AGS	
c.522del; p.Lys175ArgfsTer5	Predicted	29.8	1	0		
c.539_542dup; p.Ser181ArgfsTer19	Predicted	32.0	3	0		
c.622dup; p.Cys208LeufsTer33	Predicted	32.0	1	0		
c.625_628dup; p.Trp210SerfsTer32	Rice, 2015;	32.0	20	0	AGS	[21]
c.635del; p.Pro212HisfsTer65	ClinVar;	28.9	1	0	AGS	[18,22]
c.693dup; p.Met232HisfsTer9	LOVD;	27.8	5	0	AGS	
c.703dup; p.Val235GlyfsTer6	ClinVar;	24.6	1	0	RVCL	
c.767del; p.Thr256LysfsTer21	Predicted	22.6	6	0		
c.904del; p.Ala302ProfsTer20	Predicted	32.0	2	0		
c.907_908insT; p.Thr303IlefsTer22	Predicted	26.8	2	0		
c.912_913del; p.Tyr305TrpfsTer19	Predicted	29.2	1	0		

c.916G>T; p.Gly306Ter	Predicted	37.0	1	0

Supplementary Table 2. Summary of RVCL-S pathological studies

**Author**	**Organ**	**Evidence of Endotheliopathy**
Gulati et al. Am J Kidney Dis 2018	Kidney	Yes
Hardy et al. JNNP 2017	Brain	Yes
Braune et al. Neurol Res Pract 2024	Brain/autopsy	Yes
Khonde et al. Hum Pathol 2023	Liver	Yes
Komaki et al. Rinsho Shinkeigaku 2018	Kidney	Yes
Macaron et al. Mult Scler Relat Disord 2018	Brain	Yes
Raynowska et al. Neurology 2018	Brain	Yes
Saito et al. J Neuropathol Exp Neurol 2019	Autopsy	Yes
Stam et al. Brain 2016	Brain, Eye, Kidney	Yes
Tsubata et al. CEN Case Rep 2018	Kidney	Yes
Xie et al. Orphanet J Rare Dis 2021	Brain	No (Insufficient images)
Yan et al. BMC Neurol 2021	Brain	No (Insufficient images)

Supplementary References

1. Griffanti, L. *et al.* BIANCA (Brain Intensity AbNormality Classification Algorithm): A new tool for automated segmentation of white matter hyperintensities. *Neuroimage* **141**, 191–205 (2016).

2. Zhang, Y., Brady, M. & Smith, S. Segmentation of brain MR images through a hidden Markov random field model and the expectation-maximization algorithm. *IEEE Trans Med Imaging* **20**, 45–57 (2001).

3. Smith, S. M. *et al.* Accurate, robust, and automated longitudinal and cross-sectional brain change analysis. *Neuroimage* **17**, 479–489 (2002).

4. Fokkema, I. F. A. C. *et al.* The LOVD3 platform: efficient genome-wide sharing of genetic variants. *European Journal of Human Genetics 2021 29:12* **29**, 1796–1803 (2021).

5. Richards, S. *et al.* Standards and Guidelines for the Interpretation of Sequence Variants: A Joint Consensus Recommendation of the American College of Medical Genetics and Genomics and the Association for Molecular Pathology. *Genet Med* **17**, 405 (2015).

6. Bertucci, T. *et al.* Direct differentiation of human pluripotent stem cells into vascular network along with supporting mural cells. *APL Bioeng* **7**, (2023).

7. Chauvin, S. D. *et al.* Inherited C-terminal TREX1 variants disrupt homology-directed repair to cause senescence and DNA damage phenotypes in Drosophila, mice, and humans. *Nat Commun* **15**, 4696 (2024).

8. Alexandre, Y. O. & Mueller, S. N. An optimized protocol for the isolation of rare stromal cell populations from the mouse spleen. *STAR Protoc* **3**, (2022).

9. Seeker, L. A. *et al.* Brain matters: unveiling the distinct contributions of region, age, and sex to glia diversity and CNS function. *Acta Neuropathol Commun* **11**, (2023).

10. Guo, H. & Li, J. scSorter: assigning cells to known cell types according to marker genes. *Genome Biol* **22**, (2021).

11. Quick, S. *et al.* Loss of the heterogeneous expression of flippase ATP11B leads to cerebral small vessel disease in a normotensive rat model. *Acta Neuropathol* **144**, (2022).

12. Zhang, Y. *et al.* Purification and Characterization of Progenitor and Mature Human Astrocytes Reveals Transcriptional and Functional Differences with Mouse. *Neuron* **89**, 37–53 (2016).

13. Zhang, Y. *et al.* An RNA-Sequencing Transcriptome and Splicing Database of Glia, Neurons, and Vascular Cells of the Cerebral Cortex. *Journal of Neuroscience* **34**, 11929–11947 (2014).

14. Rodero, M. P. *et al.* Detection of interferon alpha protein reveals differential levels and cellular sources in disease. *J Exp Med* **214**, 1547–1555 (2017).

15. The brain microvasculature is a primary mediator of interferon-α neurotoxicity in human cerebral interferonopathies. *Immunity* (2024) doi:10.1016/J.IMMUNI.2024.05.017.

16. Kaminow, B., Yunusov, D. & Dobin, A. STARsolo: accurate, fast and versatile mapping/quantification of single-cell and single-nucleus RNA-seq data. *bioRxiv* 2021.05.05.442755 (2021) doi:10.1101/2021.05.05.442755.

17. Crow, Y. J. *et al.* Mutations in the gene encoding the 3'-5' DNA exonuclease TREX1 cause Aicardi-Goutières syndrome at the AGS1 locus. *Nat Genet* **38**, 917–920 (2006).

18. Ramantani, G. *et al.* Expanding the phenotypic spectrum of lupus erythematosus in Aicardi-Goutières syndrome. *Arthritis Rheum* **62**, 1469–1477 (2010).

19. Rice, G. I. *et al.* Assessment of interferon-related biomarkers in Aicardi-Goutières syndrome associated with mutations in TREX1, RNASEH2A, RNASEH2B, RNASEH2C, SAMHD1, and ADAR: a case-control study. *Lancet Neurol* **12**, 1159–1169 (2013).

20. Soong, B. W. *et al.* A homozygous NOTCH3 mutation p.R544C and a heterozygous TREX1 variant p.C99MfsX3 in a family with hereditary small vessel disease of the brain. *J Chin Med Assoc* **76**, 319–324 (2013).

21. Rice, G. *et al.* Clinical and molecular phenotype of Aicardi-Goutieres syndrome. *Am J Hum Genet* **81**, 713–725 (2007).

22. Lee-Kirsch, M. A. *et al.* Mutations in the gene encoding the 3'-5' DNA exonuclease TREX1 are associated with systemic lupus erythematosus. *Nat Genet* **39**, 1065–1067 (2007).
